# Meta-Analysis of Dilated Cardiomyopathy Using Cardiac RNA-Seq Transcriptomic Datasets

**DOI:** 10.3390/genes11010060

**Published:** 2020-01-04

**Authors:** Ahmad Alimadadi, Patricia B. Munroe, Bina Joe, Xi Cheng

**Affiliations:** 1 Department of Physiology and Pharmacology, University of Toledo College of Medicine and Life Sciences, Toledo, OH 43614, USA; ahmad.alimadadi@rockets.utoledo.edu (A.A.); bina.joe@utoledo.edu (B.J.); 2 Bioinformatics Program, University of Toledo College of Medicine and Life Sciences, Toledo, OH 43614, USA; 3 Clinical Pharmacology, William Harvey Research Institute, Barts and The London School of Medicine and Dentistry, Queen Mary University of London, London EC1M 6BQ, UK; p.b.munroe@qmul.ac.uk; 4 National Institute of Health Research Barts Cardiovascular Biomedical Research Centre, Barts and the London School of Medicine and Dentistry, Queen Mary University of London, London EC1M 6BQ, UK

**Keywords:** dilated cardiomyopathy, heat failure, meta-analysis, RNA-seq, human

## Abstract

Dilated cardiomyopathy (DCM) is one of the most common causes of heart failure. Several studies have used RNA-sequencing (RNA-seq) to profile differentially expressed genes (DEGs) associated with DCM. In this study, we aimed to profile gene expression signatures and identify novel genes associated with DCM through a quantitative meta-analysis of three publicly available RNA-seq studies using human left ventricle tissues from 41 DCM cases and 21 control samples. Our meta-analysis identified 789 DEGs including 581 downregulated and 208 upregulated genes. Several DCM-related genes previously reported, including *MYH6*, *CKM*, *NKX2–5* and *ATP2A2*, were among the top 50 DEGs. Our meta-analysis also identified 39 new DEGs that were not detected using those individual RNA-seq datasets. Some of those genes, including *PTH1R*, *ADAM15* and *S100A4*, confirmed previous reports of associations with cardiovascular functions. Using DEGs from this meta-analysis, the Ingenuity Pathway Analysis (IPA) identified five activated toxicity pathways, including failure of heart as the most significant pathway. Among the upstream regulators, *SMARCA4* was downregulated and prioritized by IPA as the top affected upstream regulator for several DCM-related genes. To our knowledge, this study is the first to perform a transcriptomic meta-analysis for clinical DCM using RNA-seq datasets. Overall, our meta-analysis successfully identified a core set of genes associated with DCM.

## 1. Introduction

Cardiovascular diseases are the leading cause of death in the United States and accounted for 840,000 deaths in 2016 [1]. Heart failure is among the cardiovascular diseases with not only a high risk of death but also with other adverse symptoms along with poor quality of life. It limits daily physical and social activities and leads to various physical and emotional distress [2]. Dilated cardiomyopathy (DCM) is one of the most common causes of heart failure, with an estimated prevalence of 1 in 2500 individuals [3]. DCM is an irreversible form of heart muscle disease and is characterized as left ventricle enlargement and systolic dysfunction. Decline in left ventricle contractile function can cause inefficient blood pumping and heart weakness, eventually leading to sudden or heart failure-related death [3].

Genetic mutations in cytoskeletal/sarcolemmal, nuclear envelope, sarcomere and transcriptional coactivator genes can cause DCM [3]. More than 20 genomic loci and genes were reported to contribute to familial DCM accounting for 20% to 35% of DCM cases [3]. Therefore, the identification of genes and their genetic variants for DCM is important and urgently needed. Advances in next-generation sequencing have led to easier and faster discovery of biomarkers for DCM. Several genes responsible for DCM, including *MYH7*, *TNNT2*, *LMNA*, *DES*, *TTN*, *PLN*, *ACTC1*, *SCN5A*, *NKX2-5* and *TBX5*, have been identified for clinical diagnosis and evaluation of genetic susceptibility [4,5,6]. RNA-seq, as a revolutionary tool in transcriptomics, enables biomarker identification by profiling differentially expressed genes (DEGs) associated with a specific disease. However, different RNA-seq studies of the same disease show inconsistent gene expression patterns and discordant results due to different sample collection and sequencing protocols and inconsistent data analysis strategies [7]. Systematic and quantitative combined analysis of multiple RNA-seq studies using a meta-analysis approach could largely eliminate the inconsistency in individual studies by increasing the sample size and statistical power for enlisting more robust disease-associated genes [8].

In this study, we performed a quantitative meta-analysis of three independent RNA-seq studies using human left ventricle tissues to identify robust candidate biomarkers for DCM. A total number of 41 DCM and 21 non-failing (NF) samples were analyzed. To our knowledge, this study is the first to perform an RNA-seq meta-analysis in the field of clinical DCM.

## 2. Materials and Methods

NCBI GEO [9] database (https://www.ncbi.nlm.nih.gov/geo/) was queried for RNA-seq studies of dilated cardiomyopathy as summarized in Table 1. The clinical information of DCM patients and their controls has been reported in Study_1 [10], Study_2 [11] and Study_3 [12]. Only studies using tissue samples obtained from human left ventricle were included in our meta-analysis.

**Figure 1:**
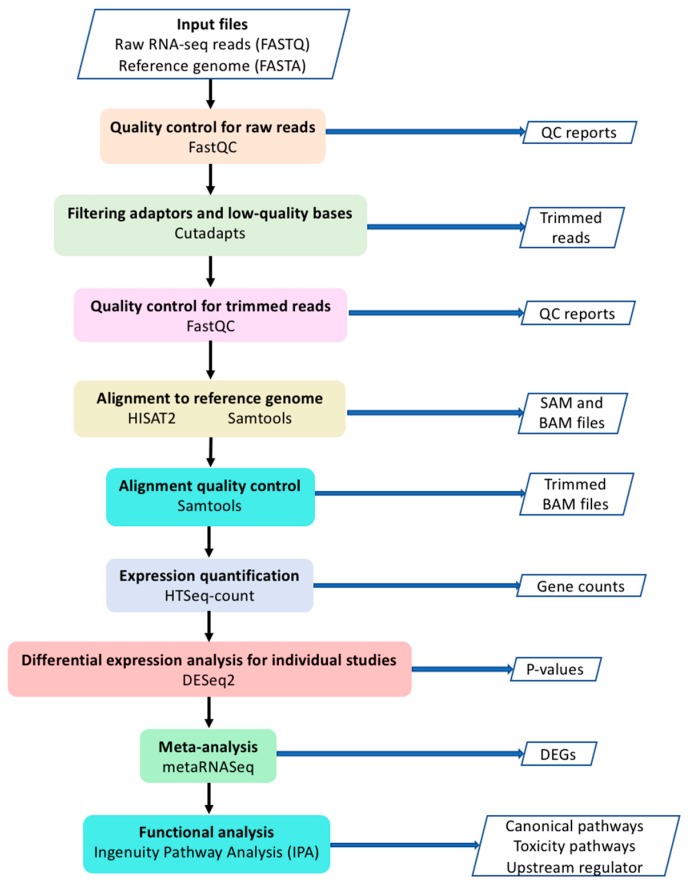
The workflow of the meta-analysis.

The workflow of our current study is shown in Figure 1. Raw data (FASTQ files) were downloaded from European Nucleotide Archive website (https://www.ebi.ac.uk/ena). Quality control (QC) for raw reads was performed using FastQC [16]. Adaptors and low-quality bases (Phred quality score < 10) were filtered by Cutadapt [17]. Trimmed reads were aligned to human reference genome (GRCh38) using HISAT2 software [18]. Samtools was used to sort the sequences and convert the SAM file to BAM file [19]. Only uniquely mapped reads were used for expression quantification with HTSeq-count [20]. Differential expression analysis for individual studies was performed using DESeq2 package [21]. Genes with low read counts were filtered out based on the mean of normalized counts as the filter statistic using independent filtering function in DESeq2 with default parameters. MetaRNASeq [7] package was used to perform Fisher’s combined probability test for meta-analysis. In this method, *p*-values for each gene from individual studies are combined through the following formula [7,22].

$$F_{g}=-2\sum_{s=1}^{s}\mathrm{In}(P_{gs})$$

When *p*-values are independent, *F*_g_ has a Chi-squared distribution with 2*S* degrees of freedom. Smaller *p*-values result in larger *F*_g_ and null hypothesis rejection. Where *P*_gs_ is the raw *p*-value obtained for gene g in a differential analysis for study *S*. *p*-values were adjusted for Benjamini–Hochberg false discovery rate (FDR). Adjusted *p*-value smaller than 0.05 was considered as statistically significant. Genes with inconsistent expression directions (upregulated or downregulated) among individual studies were removed from the DEGs list.

Using DEGs from meta-analysis, the Ingenuity Pathway Analysis (IPA, Qiagen, Redwood City, California, USA) was performed to investigate enriched canonical pathways, toxicity functions (IPA-Tox) and upstream regulators [23]. IPA-Tox uses the current knowledge to link DEGs to clinical pathological endpoints. IPA upstream regulator tool also uses current knowledge to identify the upstream transcriptional regulators which explain the observed gene expression changes in a dataset. A Venn diagram of the number of DEGs from individual studies and the meta-analysis was generated using the VennDiagram package [24] in R. The Ohio Supercomputer Center (OSC) was used to perform the compute-intensive tasks [25].

## 3. Results

### 3.1. Datasets Analyzed in This Study

Based on our search criteria (RNA-seq, heart failure, dilated cardiomyopathy and human), three RNA-seq datasets (GEO accession numbers GSE116250, GSE57344, and GSE71613) were included in our study. Total numbers of DCM and NF samples were 41 and 21, respectively. The summary of the datasets is shown in Table 1.

### 3.2. Differentially Expressed Genes

The human gene set, Homo_sapiens.GRCh38.96.gtf, including a total of 58,884 coding and non-coding genes, was used for expression quantification. Our meta-analysis identified 789 DEGs including 581 downregulated and 208 upregulated genes at a false discovery rate (FDR) < 0.05 (Supplementary Table S1). The top 50 DEGs sorted by *p*-value are shown in Table 2.

**Figure 2:**
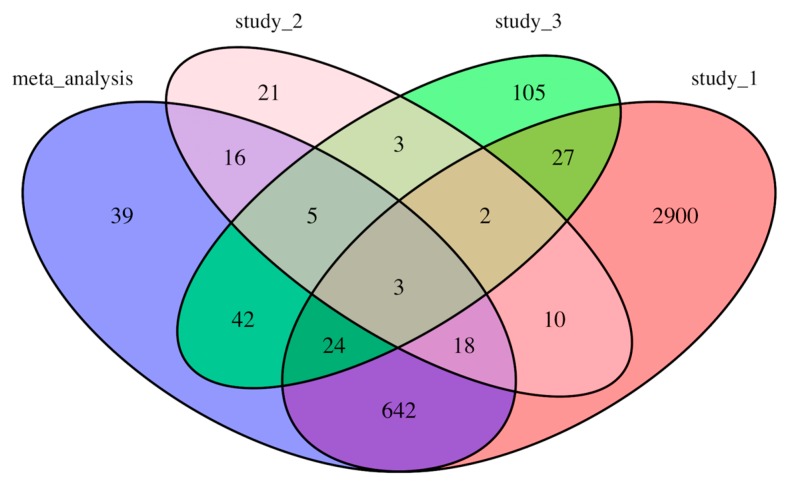
Venn diagram showing the number of differential expressed genes from individual studies and meta-analysis.

The counts of the common genes shared among individual studies (results from our analyses) and meta-analysis are shown in a Venn diagram (Figure 2). There are 39 DEGs that were only identified through the meta-analysis but not in individual analyses (Table 3). Interestingly, a significant number of DEGs from each individual study did not appear in other studies or meta-analysis. For example, 2900 genes out of total 3626 DEGs in Study_1 were not identified to be differentially expressed in other studies or in our meta-analysis (Figure 2). Only three genes, *TUBA3D*, *LCN10*, and *NPR3*, were common among all three individual analyses and the meta-analysis (Figure 2).

### 3.3. Toxicity Pathway Analysis

**Figure 3:**
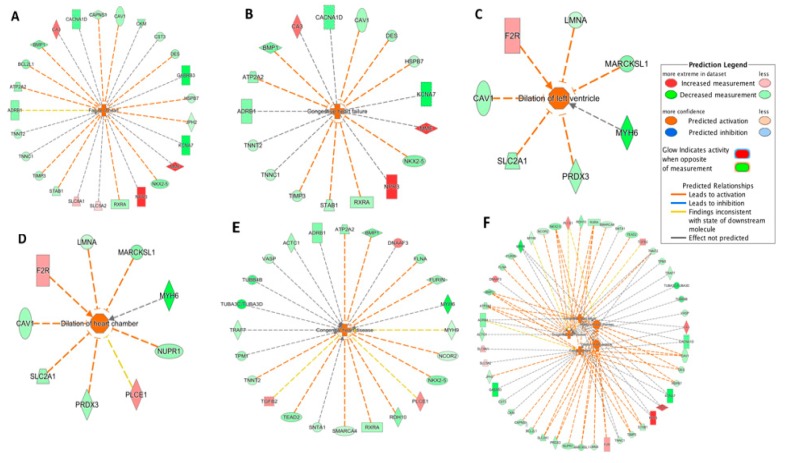
Toxicity pathways significantly activated in DCM through IPA-Tox: (**A**) failure of heart; (**B**) congestive heart failure; (**C**) dilation of left ventricle; (**D**) dilation of heart chamber; (**E**) congestive heart disease; (**F**) integrated toxicity pathways. As an example, in Figure 3A, the *DES* gene was downregulated as indicated by the green color and the downregulation of *DES* further promoted (indicated by the orange dash line) the activation of failure of heart, as indicated by the orange color. Other indicators are explained in the Prediction Legend section.

The IPA-Tox tool was used to identify enriched toxicity pathways. Out of 184 significant toxicity pathways (*p* < 0.05), only five pathways were identified as activated (z-score > 2.0). These were failure of heart, congestive heart failure, dilation of left ventricle, dilation of heart chamber and congenital heart disease. Responsible DEGs for each toxicity pathway and their direction of change are shown in the Figure 3A–E. Failure of heart and congestive heart failure had the highest activation z-score (3.06 and 2.96, respectively). The five pathways and their corresponding DEGs were further integrated (Figure 3F). Among the 39 DEGs from the integrated network (Figure 3F), 8 DEGs were shared in three or more pathways. Interestingly, these eight DEGs were all downregulated in the DCM group (Table 4). *ATP2A2*, *MYH6*, and *NKX2-5* were among the top 50 DEGs (Table 2).

### 3.4. Upstream Regulator Analysis

Upstream regulator analysis was performed through IPA. Differential expression data from the meta-analysis were used for constructing upstream regulator-targeted genes networks. These regulators could be transcription factors, non-coding RNA, enzymes or other molecules. A total of 876 potential upstream regulators were identified as significant (*p*-value of overlap < 0.05). Further, 38 of those regulators were differentially expressed in our dataset (29: downregulated; nine: upregulated in DCM) (Supplementary Table S2).

**Figure 4:**
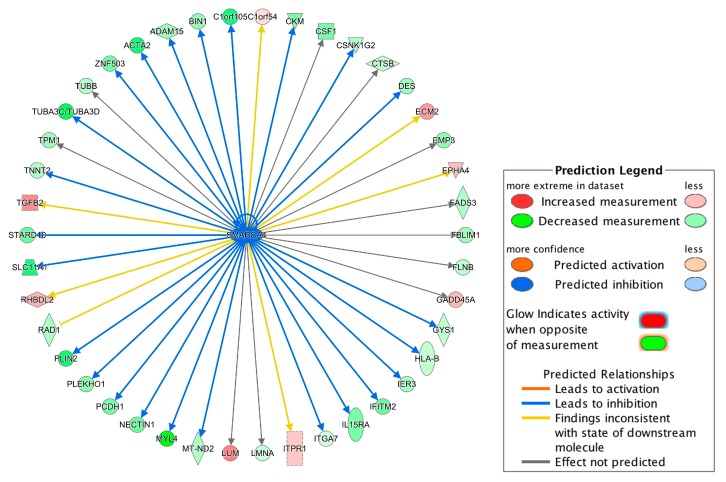
Network of the upstream regulator *SMARCA4* and its targeted genes. The network was constructed using the differentially expressed genes identified in our meta-analysis. For example, the downregulation of *SMARCA4* led to the inhibition (indicated by the blue arrow line) of the *DES* expression and the downregulation of *DES* is indicated by the green color. Other indicators are explained in the Prediction Legend section.

Among the 38 regulators, *SMARCA4* (also known as *Brg1*) had the highest absolute prediction score (z-score = −3.00) and it was downregulated in the DCM group (Supplementary Table S2). Figure 4 summarized *SMARCA4*-regulated genes. Interestingly, the downregulation of *SMARCA4* resulted in *DES* and *TNNT2* downregulation (Figure 4), which could further promote failure of heat, congestive heart failure and congestive heart disease based on the IPA-Tox analysis (Figure 3). Therefore, *SMARCA4* is a promising upstream regulator candidate regulating multiple downstream genes contributing to heart failure-related phenotypes.

### 3.5. Canonical Pathway Analysis

**Figure 5:**
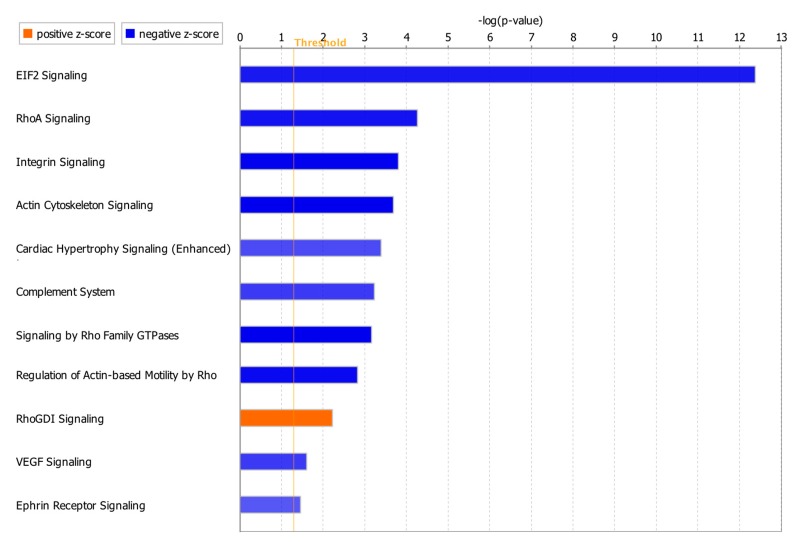
Significant canonical pathways with absolute z-score > 2.0. The z-score represents the activation or inhibition state of a canonical pathway. A z-score greater than 2.0 was considered as significantly activated. A z-score smaller than −2.0 was considered as significantly inhibited.

Canonical pathway analysis through IPA identified 96 significant pathways (*p*-value < 0.05). Considering z-score with more than 2.0 for significant activation or less than −2.0 for significant inhibition status in the pathway analysis, only 11 of the total 96 pathways had absolute z-score more than 2.0 with 10 pathways as significantly inhibited and one pathway as significantly activated (Figure 5). DEGs involved in each pathway are summarized in Supplementary Table S3. *NKX2-5*, *CAV1* and *ATP2A2* in the integrin signaling and cardiac hypertrophy signaling (enhanced) pathways (Supplementary Table S3) were also involved in the toxicity pathways such as failure of heart, congenital heart disease, congestive heart failure, dilation of heart chamber and dilation of left ventricle (Figure 3, Table 4). *IL15RA*, *MAP3K6* and *CD14* were among the genes which were identified only in our meta-analysis, but not in the individual studies (Table 3).

## 4. Discussion

In this study, we performed a quantitative meta-analysis of three independent RNA-seq studies using human left ventricle tissues to profile gene expression signatures and identify novel genes associated with DCM. To better integrate the RNA-seq results from different studies, we applied a consistent bioinformatics pipeline (Figure 1) to analyze the raw RNA-seq data (FASTQ files) from the three independent studies in which three different pipelines were used [10,11,12]. Among a total of 58,884 genes used for expression quantification, 789 genes were identified as differentially expressed in meta-analysis including 581 downregulated and 208 upregulated genes (Supplementary Table S1). Only three genes, *LCN10*, *TUBA3D* and *NPR3*, were common between all three individual analyses and the meta-analysis (Figure 2). Interestingly, these three genes were among the genes with the high fold change in our analysis (log_2_FC = −2.64, −1.75 and 2.14, respectively) (Supplementary Table S1). *LCN10* is a member of the lipocalin family that generally binds to small hydrophobic ligands and transport them to specific cells [26]. *LCN10* has been shown to be significantly downregulated in patients with right ventricular heart failure [27]. *TUBA3D*, also known as *TUBA2*, encodes a member of the α tubulin family which is a major component of microtubules. *TUBA3D* along with other genes encoding microtubule subunits has been reported previously to be downregulated in DCM patients [28]. *NPR3* (also known as *NPR-C*) encodes one of three receptors of natriuretic peptide which are small peptides responsible for regulating blood volume and pressure [29]. Several variations of *NPR3* have been previously shown to be related to essential hypertension and coronary artery disease [30,31]. Follow-up functional studies are needed to investigate the mechanisms by which these genes contribute to the development of cardiomyopathy. 

Specific alleles or change in expression levels of several genes from the top 50 DEG list (Table 2) such as *MYH6* [32], *CKM* [33], *NKX2-5* [34,35], and *ATP2A2* [36] have been previously reported to be associated with DCM. *MYH6* encoding α heavy chain subunit of cardiac myosin was downregulated in the DCM group in our dataset. Mutations in *MYH6* have been reported in patients with both dilated and hypertrophic phenotypes of cardiomyopathy [32]. Inhibition of *CKM*, a cytoplasmic enzyme, which is a serum marker for myocardial infarction and contributes to energy homeostasis, has been also reported to inhibit cardiac contractility and contractility of muscle [29,33]. *NKX2-5*, which encodes a homeobox-containing transcription factor, was downregulated in our dataset [29]. Mutation in this gene could affect heart formation and development and has been reported in DCM [34,35]. *ATP2A2*, an ATPase enzyme that regulates the level of calcium ions inside the cells, is found in the sarcoplasmic reticulum involved in muscle contraction and relaxation by releasing and storing calcium ions [29]. Downregulation of *ATP2A2* has been reported during human end-stage heart failure and has been suggested to be responsible for change in Ca2+ movements and myocardial relaxation [36].

Our meta-analysis identified 39 unique DEGs that were not identified as DEGs in individual analyses (Table 3), indicating that our meta-analysis further increased the statistical power for identifying new disease-associated genes, which has been reported as an advantage of the meta-analysis approach [7,8]. Some of these genes, including *PTH1R*, *S100A4* and *ADAM15*, have been reported to be differentially expressed in DCM or other types of cardiomyopathies. *PTH1R* encodes a protein from G-protein coupled receptor family 2, which is a receptor for parathyroid hormone (*PTH*) and for parathyroid hormone-like hormone (*PTHLH*) [29]. This gene was downregulated in our study. Although no report was found for direct association between *PTH1R* and DCM, *PTH* has direct effects on heart and calcium homeostasis, which could impact cardiovascular system [37]. Other studies have also observed the downregulation of *S100A4* and upregulation of *ADAM15* in failing heart in human [38,39]. However, *ADAM15* was downregulated in our study, suggesting a complicated pathophysiological role of *ADAM15* in heart failure. 

For further understanding physiological processes involved in DCM, we performed Ingenuity Pathway Analysis (IPA, Qiagen). Five toxicity pathways, including failure of heart (with the highest activation z-score), were identified as significantly activated in the DCM group. Interestingly, all these activated toxicity pathways were related to heart failure and cardiac dilation, demonstrating that the identified DEGs in our meta-analysis are significant to capture the progression of heart failure.

Among the upstream regulators, *SMARCA4* with the highest absolute prediction score (z-score = −3.00) was downregulated in the DCM group and was predicted to target 42 genes (Figure 4). *SMARCA4*, also known as *BRG1*, is a member of the SWI/SNF family of proteins with helicase and ATPase activities and is known for regulating the transcription of several genes by altering the chromatin structure [29]. Among the genes involved in progression of toxicity pathways (Figure 3), *DES* and *TNNT2* are targeted by *SMARCA4* (Figure 4) and the downregulation of *SMARCA4* resulted in the downregulation of *DES* and *TNNT2*. The evidence shows that chromatin remodeling is required for *DES* expression [40] and inhibition or lack of *DES* could lead to idiopathic DCM [41]. Inhibition of *TNNT2* was also previously reported to lead to left ventricular dilation and impaired contractility [42].

Canonical pathway analysis showed that 11 pathways were affected with ten significantly inhibited and one significantly activated (Figure 5). Actin cytoskeleton signaling, Ras homolog family member A (RhoA) signaling, integrin signaling, cardiac hypertrophy signaling (enhanced) and vascular endothelial growth factor (VEGF) signaling pathways have been previously reported to be related to cardiac functions [43,44,45,46,47]. Change in actin cytoskeleton has been considered for the regulation of cardiomyocyte [43]. RhoA in cardiomyocytes can have both detrimental and beneficial effects on the heart as it has been reported to prevent progression of dilation and heart failure, but also promote the cardiac fibrosis [44]. Uncontrolled integrin activation could also result in contractile dysfunction and arrhythmias [45]. Downregulation of VEGF has been observed in dilated cardiomyopathy and heart failure [46]. Interestingly, several genes including *NKX2-5*, *ATP2A2* and *CAV1* involved in these pathways were also found in toxicity pathways such as failure of heart, congenital heart disease, congestive heart failure, dilation of heart chamber and dilation of left ventricle. Roles of *NKX2-5* and *ATP2A2* in developing cardiac diseases were discussed above. Loss of *CAV-1* gene expression in mice has been also reported to cause the progression of hypertrophic cardiomyopathy and sudden cardiac death syndrome [48]. Overall, our meta-analysis successfully identified a core set of genes associated with DCM including well-known cardiomyopathy biomarkers as well as newly identified genes. This set of genes from our meta-analysis will be provided as more robust candidates for DCM compared to the genes profiled from individual studies. Our meta-analysis further provided the DCM-associated genes as potential diagnostic biomarkers and therapeutic targets for future clinical application. For most of the new DEGs identified in our study, there is no report of a direct relationship with DCM. Thus, future research on these new DEGs is needed to validate their physiological roles in DCM and further understand their molecular mechanisms in cardiovascular pathophysiology, especially cardiomyopathies. 

## Figures and Tables

**Table 1:** Summary of GEO datasets for the meta-analysis.

Study	Dataset	Platform	Sample Size	Tissue
Study_1	GSE116250 [13]	Illumina HiSeq 2500	37 DCM 14 NF	left ventricle
Study_2	GSE57344 [14]	Illumina HiSeq 2000	2 DCM 3 NF	left ventricle
Study_3	GSE71613 [15]	Illumina HiSeq 2000	2 DCM 4 NF	left ventricle

**Table 2:** Top 50 differential expressed genes identified in meta-analysis of DCM vs. NF.

Ensembl_ID	Gene_Symbol	Adj_P ^1^	Average_Log_2_FCm ^2^	Effectm ^3^
ENSG00000076351	*SLC46A1*	0.00E + 00	0.37	Up
ENSG00000113389	*NPR3*	0.00E + 00	2.14	Up
ENSG00000126106	*TMEM53*	0.00E + 00	−0.60	Down
ENSG00000176293	*ZNF135*	0.00E + 00	0.37	Up
ENSG00000177575	*CD163*	0.00E + 00	−1.57	Down
ENSG00000189060	*H1F0*	0.00E + 00	−1.01	Down
ENSG00000227495	*AC004771.1*	0.00E + 00	−0.40	Down
ENSG00000263986	*AC087393.2*	0.00E + 00	0.49	Up
ENSG00000179526	*SHARPIN*	1.68E−13	−0.54	Down
ENSG00000197616	*MYH6*	1.68E−13	−1.85	Down
ENSG00000157388	*CACNA1D*	3.03E−13	−1.10	Down
ENSG00000229867	*STEAP3-AS1*	5.87E−13	−0.71	Down
ENSG00000167646	*DNAAF3*	1.28E−12	1.30	Up
ENSG00000170448	*NFXL1*	7.58E−12	−2.33	Down
ENSG00000186187	*ZNRF1*	2.44E−11	−0.56	Down
ENSG00000259661	*AC068831.4*	2.57E−11	−0.59	Down
ENSG00000142156	*COL6A1*	3.39E−11	−0.78	Down
ENSG00000235910	*APOA1-AS*	4.28E−11	0.90	Up
ENSG00000109099	*PMP22*	4.81E−11	−0.86	Down
ENSG00000163220	*S100A9*	6.09E−11	−2.13	Down
ENSG00000174437	*ATP2A2*	8.49E−11	−0.83	Down
ENSG00000175221	*MED16*	1.06E−10	−0.83	Down
ENSG00000198624	*CCDC69*	1.60E−10	−1.04	Down
ENSG00000133800	*LYVE1*	1.72E−10	−2.06	Down
ENSG00000267092	*AC027307.1*	1.85E−10	0.63	Up
ENSG00000129250	*KIF1C*	2.27E−10	−0.61	Down
ENSG00000256469	*AP002383.2*	2.84E−10	0.82	Up
ENSG00000233098	*CCDC144NL-AS1*	3.19E−10	0.37	Up
ENSG00000105679	*GAPDHS*	3.53E−10	0.65	Up
ENSG00000156463	*SH3RF2*	3.66E−10	−1.05	Down
ENSG00000210127	*MT-TA*	3.66E−10	−1.00	Down
ENSG00000183072	*NKX2-5*	9.88E−10	−0.96	Down
ENSG00000125733	*TRIP10*	1.08E−09	−0.88	Down
ENSG00000141905	*NFIC*	1.11E−09	−0.61	Down
ENSG00000135447	*PPP1R1A*	1.14E−09	−1.00	Down
ENSG00000107796	*ACTA2*	1.35E−09	−1.38	Down
ENSG00000140675	*SLC5A2*	1.75E−09	0.41	Up
ENSG00000101187	*SLCO4A1*	2.57E−09	−1.91	Down
ENSG00000103710	*RASL12*	3.61E−09	−1.34	Down
ENSG00000155659	*VSIG4*	3.85E−09	−2.04	Down
ENSG00000253549	*CA3-AS1*	4.04E−09	2.41	Up
ENSG00000105698	*USF2*	5.02E−09	−0.50	Down
ENSG00000196642	*RABL6*	5.44E−09	−0.52	Down
ENSG00000122034	*GTF3A*	8.93E−09	−0.62	Down
ENSG00000235790	*AC114488.2*	9.04E−09	0.83	Up
ENSG00000104879	*CKM*	1.05E−08	−0.95	Down
ENSG00000114867	*EIF4G1*	1.21E−08	−0.80	Down
ENSG00000260469	*INSYN1-AS1*	1.33E−08	−0.96	Down
ENSG00000260755	*AC010542.2*	1.49E−08	0.38	Up
ENSG00000257453	*AC011611.3*	1.60E−08	1.39	Up

^1^: FDR-adjusted *p*-value, “E” represents “times ten raised to the power of”; ^2^: Average of log_2_FC from individual studies; ^3^: ‘Up’ or ‘Down’ indicates whether the gene was upregulated or downregulated.

**Table 3:** Differential expressed genes newly identified in the meta-analysis of DCM vs. NF.

Ensembl_ID	Gene_Symbol	Adj_p ^1^	Average_Log_2_FC ^2^	Effect ^3^
ENSG00000158859	*ADAMTS4*	1.44E−03	−1.15	Down
ENSG00000185201	*IFITM2*	6.57E−03	−1.03	Down
ENSG00000162551	*ALPL*	8.13E−03	−1.28	Down
ENSG00000151365	*THRSP*	8.81E−03	1.71	Up
ENSG00000142733	*MAP3K6*	8.95E−03	−0.91	Down
ENSG00000158246	*TENT5B*	1.03E−02	−1.35	Down
ENSG00000087495	*PHACTR3*	1.38E−02	−1.57	Down
ENSG00000132205	*EMILIN2*	1.53E−02	−0.95	Down
ENSG00000103316	*CRYM*	1.65E−02	0.88	Up
ENSG00000224934	*AL391684.1*	1.65E−02	0.73	Up
ENSG00000198542	*ITGBL1*	1.68E−02	1.02	Up
ENSG00000174429	*ABRA*	1.72E−02	−1.02	Down
ENSG00000172935	*MRGPRF*	1.76E−02	−0.98	Down
ENSG00000196154	*S100A4*	2.03E−02	−1.09	Down
ENSG00000131386	*GALNT15*	2.08E−02	−1.16	Down
ENSG00000255750	*AC022509.1*	2.12E−02	1.14	Up
ENSG00000198517	*MAFK*	2.48E−02	0.68	Up
ENSG00000143537	*ADAM15*	2.51E−02	−0.77	Down
ENSG00000134470	*IL15RA*	2.52E−02	−0.92	Down
ENSG00000170458	*CD14*	2.75E−02	−0.88	Down
ENSG00000242396	*AC096536.2*	3.26E−02	−1.14	Down
ENSG00000203883	*SOX18*	3.39E−02	−0.74	Down
ENSG00000132481	*AC087289.1*	3.44E−02	−0.74	Down
ENSG00000162458	*FBLIM1*	3.54E−02	−0.78	Down
ENSG00000106809	*OGN*	3.61E−02	1.18	Up
ENSG00000196923	*PDLIM7*	3.76E−02	−0.98	Down
ENSG00000167797	*CDK2AP2*	3.94E−02	−0.81	Down
ENSG00000160801	*PTH1R*	4.02E−02	−0.87	Down
ENSG00000147155	*EBP*	4.05E−02	−1.18	Down
ENSG00000177679	*SRRM3*	4.18E−02	−1.07	Down
ENSG00000106823	*ECM2*	4.22E−02	1.00	Up
ENSG00000173272	*MZT2A*	4.33E−02	−0.66	Down
ENSG00000106819	*ASPN*	4.40E−02	1.27	Up
ENSG00000180999	*C1orf105*	4.76E−02	−1.41	Down
ENSG00000175287	*PHYHD1*	4.85E−02	−0.87	Down
ENSG00000279296	*PRAL*	4.88E−02	0.50	Up
ENSG00000187955	*COL14A1*	4.90E-02	0.93	Up
ENSG00000134684	*YARS*	4.91E-02	-0.68	Down
ENSG00000228804	*AC072022.1*	4.96E-02	-0.71	Down

^1^: FDR-adjusted *p*-value, “E” represents “times ten raised to the power of”; ^2^: Average of log_2_FC from individual studies; ^3^: ‘Up’ or ‘Down’ indicates whether the gene was upregulated or downregulated.

**Table 4:** DEGs common among three or more toxicity pathways through IPA-Tox analysis.

DEG	Log_2_FC ^1^	Tox Pathway
*ADRB1*	−0.90	Failure of heart
		Congenital heart disease
		Congestive heart failure
*ATP2A2*	−0.83	Failure of heart
		Congenital heart disease
		Congestive heart failure
*BMP1*	−1.04	Failure of heart
		Congenital heart disease
		Congestive heart failure
*CAV1*	−0.72	Failure of heart
		Congestive heart failure
		Dilation of heart chamber
		Dilation of left ventricle
*MYH6*	−1.85	Congenital heart disease
		Dilation of heart chamber
		Dilation of left ventricle
*NKX2-5*	−0.96	Failure of heart
		Congenital heart disease
		Congestive heart failure
*RXRA*	−0.68	Failure of heart
		Congenital heart disease
		Congestive heart failure
*TNNT2*	−0.62	Failure of heart
		Congenital heart disease
		Congestive heart failure

^1^: Average of log_2_FC from individual studies.

## Supplementary Materials

The following are available online at https://www.mdpi.com/2073-4425/11/1/60/s1. Table S1: A complete list of differential expressed genes identified in meta-analysis of DCM vs NF. Table S2: Differentially expressed upstream regulators and their target genes. Table S3: Significant canonical pathways with absolute z-score > 2.0 and the involved genes.
