# Supplementary material for: Meta-Analysis of Dilated Cardiomyopathy Using Cardiac RNA-Seq Transcriptomic Datasets
Source: Genes (Basel). 2020 Jan 4;11(1):60. doi: 10.3390/genes11010060 (PMC7017089; doi:10.3390/genes11010060)
Supplement: Supplementary file 1 [file genes-11-00060-s001.pdf]

Supplementary Tables

Supplementary Table S1. A complete list of differential expressed genes identified in meta-analysis of DCM vs NF.

Ensembl_ID	gene_symbol	DE_1[1]	DE_2[1]	DE_3[1]	adj_p[2]	average_log$_2$FC[3]	effect[4]
ENSG00000076351	*SLC46A1*	Yes	No	No	0.00E+00	0.37	Up
ENSG00000113389	*NPR3*	Yes	Yes	Yes	0.00E+00	2.14	Up
ENSG00000126106	*TMEM53*	Yes	No	No	0.00E+00	-0.60	Down
ENSG00000176293	*ZNF135*	Yes	No	No	0.00E+00	0.37	Up
ENSG00000177575	*CD163*	No	Yes	No	0.00E+00	-1.57	Down
ENSG00000189060	*H1F0*	Yes	No	No	0.00E+00	-1.01	Down
ENSG00000227495	*AC004771.1*	Yes	No	No	0.00E+00	-0.40	Down
ENSG00000263986	*AC087393.2*	Yes	No	No	0.00E+00	0.49	Up
ENSG00000179526	*SHARPIN*	Yes	No	No	1.68E-13	-0.54	Down
ENSG00000197616	*MYH6*	Yes	No	No	1.68E-13	-1.85	Down
ENSG00000157388	*CACNA1D*	Yes	No	No	3.03E-13	-1.10	Down
ENSG00000229867	*STEAP3-AS1*	Yes	No	No	5.87E-13	-0.71	Down
ENSG00000167646	*DNAAF3*	Yes	No	No	1.28E-12	1.30	Up
ENSG00000170448	*NFXL1*	Yes	No	Yes	7.58E-12	-2.33	Down
ENSG00000186187	*ZNRF1*	Yes	No	No	2.44E-11	-0.56	Down
ENSG00000259661	*AC068831.4*	Yes	No	No	2.57E-11	-0.59	Down
ENSG00000142156	*COL6A1*	Yes	No	No	3.39E-11	-0.78	Down
ENSG00000235910	*APOA1-AS*	Yes	No	No	4.28E-11	0.90	Up
ENSG00000109099	*PMP22*	Yes	No	No	4.81E-11	-0.86	Down
ENSG00000163220	*S100A9*	Yes	Yes	No	6.09E-11	-2.13	Down
ENSG00000174437	*ATP2A2*	Yes	No	No	8.49E-11	-0.83	Down
ENSG00000175221	*MED16*	Yes	No	No	1.06E-10	-0.83	Down
ENSG00000198624	*CCDC69*	Yes	No	No	1.60E-10	-1.04	Down
ENSG00000133800	*LYVE1*	Yes	No	Yes	1.72E-10	-2.06	Down
ENSG00000267092	*AC027307.1*	Yes	No	No	1.85E-10	0.63	Up
ENSG00000129250	*KIF1C*	Yes	No	No	2.27E-10	-0.61	Down
ENSG00000256469	*AP002383.2*	Yes	No	No	2.84E-10	0.82	Up
ENSG00000233098	*CCDC144NL-AS1*	Yes	No	No	3.19E-10	0.37	Up
ENSG00000105679	*GAPDHS*	Yes	No	No	3.53E-10	0.65	Up
ENSG00000156463	*SH3RF2*	Yes	No	No	3.66E-10	-1.05	Down
ENSG00000210127	*MT-TA*	Yes	No	No	3.66E-10	-1.00	Down
ENSG00000183072	*NKX2-5*	Yes	No	No	9.88E-10	-0.96	Down
ENSG00000125733	*TRIP10*	Yes	No	No	1.08E-09	-0.88	Down
ENSG00000141905	*NFIC*	Yes	No	No	1.11E-09	-0.61	Down

ENSG00000135447	*PPP1R1A*	Yes	No	Yes	1.14E-09	-1.00	Down
ENSG00000107796	*ACTA2*	No	Yes	Yes	1.35E-09	-1.38	Down
ENSG00000140675	*SLC5A2*	Yes	No	No	1.75E-09	0.41	Up
ENSG00000101187	*SLCO4A1*	No	Yes	Yes	2.57E-09	-1.91	Down
ENSG00000103710	*RASL12*	Yes	No	Yes	3.61E-09	-1.34	Down
ENSG00000155659	*VSIG4*	Yes	Yes	No	3.85E-09	-2.04	Down
ENSG00000253549	*CA3-AS1*	Yes	No	Yes	4.04E-09	2.41	Up
ENSG00000105698	*USF2*	Yes	No	No	5.02E-09	-0.50	Down
ENSG00000196642	*RABL6*	Yes	No	No	5.44E-09	-0.52	Down
ENSG00000122034	*GTF3A*	Yes	No	No	8.93E-09	-0.62	Down
ENSG00000235790	*AC114488.2*	Yes	No	No	9.04E-09	0.83	Up
ENSG00000104879	*CKM*	Yes	No	No	1.05E-08	-0.95	Down
ENSG00000114867	*EIF4G1*	Yes	No	No	1.21E-08	-0.80	Down
ENSG00000260469	*INSYN1-AS1*	Yes	No	No	1.33E-08	-0.96	Down
ENSG00000260755	*AC010542.2*	Yes	No	No	1.49E-08	0.38	Up
ENSG00000257453	*AC011611.3*	Yes	Yes	No	1.60E-08	1.39	Up
ENSG00000104894	*CD37*	Yes	No	No	1.83E-08	-0.36	Down
ENSG00000272345	*AL031775.1*	Yes	No	No	2.25E-08	-0.48	Down
ENSG00000253645	*AC108863.1*	Yes	No	No	2.61E-08	0.41	Up
ENSG00000156471	*PTDSS1*	Yes	No	No	3.01E-08	-1.25	Down
ENSG00000178297	*TMPRSS9*	Yes	No	No	3.37E-08	0.48	Up
ENSG00000274021	*AC024909.1*	Yes	No	No	3.37E-08	0.98	Up
ENSG00000187837	*HIST1H1C*	Yes	No	No	3.55E-08	-0.80	Down
ENSG00000205363	*INSYN1*	Yes	No	Yes	3.96E-08	-1.30	Down
ENSG00000137331	*IER3*	Yes	No	No	4.20E-08	-0.67	Down
ENSG00000188229	*TUBB4B*	Yes	No	No	4.20E-08	-1.20	Down
ENSG00000125730	*C3*	Yes	No	No	4.63E-08	-1.39	Down
ENSG00000175084	*DES*	Yes	No	No	4.91E-08	-0.73	Down
ENSG00000224985	*AL590714.1*	Yes	No	No	5.34E-08	-0.33	Down
ENSG00000139289	*PHLDA1*	Yes	Yes	No	5.50E-08	1.28	Up
ENSG00000137818	*RPLP1*	Yes	No	No	5.71E-08	-0.78	Down
ENSG00000105058	*FAM32A*	Yes	No	No	6.51E-08	-0.85	Down
ENSG00000272288	*AL451165.2*	Yes	No	No	6.58E-08	-0.39	Down
ENSG00000189058	*APOD*	Yes	No	Yes	7.26E-08	-1.28	Down
ENSG00000163050	*COQ8A*	Yes	No	No	7.30E-08	-0.87	Down
ENSG00000101335	*MYL9*	Yes	No	No	7.31E-08	-0.88	Down
ENSG00000196924	*FLNA*	Yes	No	No	7.31E-08	-0.83	Down
ENSG00000149294	*NCAM1*	Yes	No	No	7.54E-08	-0.61	Down
ENSG00000125844	*RRBP1*	Yes	No	No	7.81E-08	-0.48	Down
ENSG00000182175	*RGMA*	Yes	No	No	8.37E-08	-1.14	Down

ENSG00000259683	*AC243562.2*	Yes	No	No	8.68E-08	0.89	Up
ENSG00000019169	*MARCO*	Yes	Yes	No	8.80E-08	-2.93	Down
ENSG00000034510	*TMSB10*	Yes	No	No	8.80E-08	-1.02	Down
ENSG00000152580	*IGSF10*	Yes	No	No	9.06E-08	1.21	Up
ENSG00000166091	*CMTM5*	Yes	Yes	No	9.23E-08	-1.52	Down
ENSG00000187922	*LCN10*	Yes	Yes	Yes	9.40E-08	-2.64	Down
ENSG00000123240	*OPTN*	Yes	No	No	1.35E-07	-0.73	Down
ENSG00000197548	*ATG7*	Yes	No	No	1.58E-07	0.26	Up
ENSG00000259488	*AC023355.1*	Yes	No	No	1.70E-07	0.42	Up
ENSG00000103023	*PRSS54*	Yes	No	No	1.79E-07	1.12	Up
ENSG00000173402	*DAG1*	Yes	No	No	1.94E-07	-0.70	Down
ENSG00000085662	*AKR1B1*	Yes	No	Yes	1.99E-07	-1.36	Down
ENSG00000140534	*TICRR*	Yes	No	No	2.18E-07	0.69	Up
ENSG00000165916	*PSMC3*	Yes	No	No	2.22E-07	-0.74	Down
ENSG00000267577	*AC010327.3*	Yes	No	No	2.24E-07	1.18	Up
ENSG00000120729	*MYOT*	Yes	Yes	No	2.25E-07	-1.08	Down
ENSG00000224195	*AC022400.1*	Yes	No	No	2.33E-07	0.50	Up
ENSG00000254254	*AC012349.1*	Yes	No	No	2.46E-07	2.00	Up
ENSG00000129538	*RNASE1*	Yes	No	No	2.68E-07	-1.15	Down
ENSG00000113369	*ARRDC3*	Yes	No	No	2.94E-07	0.97	Up
ENSG00000138193	*PLCE1*	Yes	No	Yes	3.27E-07	1.13	Up
ENSG00000141736	*ERBB2*	Yes	No	No	3.27E-07	-0.58	Down
ENSG00000140416	*TPM1*	Yes	No	No	3.28E-07	-0.74	Down
ENSG00000255252	*AL078612.1*	Yes	No	No	3.80E-07	-0.44	Down
ENSG00000184164	*CRELD2*	Yes	No	No	4.70E-07	-0.61	Down
ENSG00000166206	*GABRB3*	Yes	No	Yes	4.80E-07	-1.70	Down
ENSG00000198763	*MT-ND2*	Yes	No	No	5.34E-07	-0.61	Down
ENSG00000257653	*AC117498.1*	Yes	No	No	6.01E-07	0.49	Up
ENSG00000277258	*PCGF2*	Yes	No	No	6.62E-07	-0.40	Down
ENSG00000167996	*FTH1*	Yes	No	No	7.00E-07	-0.72	Down
ENSG00000147872	*PLIN2*	Yes	Yes	No	8.32E-07	-1.43	Down
ENSG00000161970	*RPL26*	Yes	No	No	8.36E-07	-0.77	Down
ENSG00000149716	*LTO1*	Yes	No	No	8.77E-07	0.43	Up
ENSG00000152086	*TUBA3E*	Yes	No	Yes	8.78E-07	-2.46	Down
ENSG00000173369	*C1QB*	Yes	Yes	No	8.78E-07	-1.42	Down
ENSG00000286018	*AF129075.3*	Yes	No	No	9.51E-07	-0.28	Down
ENSG00000196739	*COL27A1*	Yes	No	No	1.14E-06	-1.02	Down
ENSG00000125691	*RPL23*	Yes	No	No	1.28E-06	-0.63	Down
ENSG00000184489	*PTP4A3*	Yes	No	Yes	1.69E-06	-0.94	Down
ENSG00000159387	*IRX6*	Yes	No	No	1.71E-06	1.97	Up

ENSG00000203804	*ADAMTSL4-AS1*	No	No	Yes	1.74E-06	-0.99	Down
ENSG00000164855	*TMEM184A*	Yes	No	No	1.92E-06	0.77	Up
ENSG00000196498	*NCOR2*	Yes	No	No	1.92E-06	-0.43	Down
ENSG00000198336	*MYL4*	Yes	No	Yes	1.93E-06	-2.60	Down
ENSG00000198157	*HMGN5*	Yes	No	No	2.25E-06	-0.92	Down
ENSG00000257950	*P2RX5-TAX1BP3*	Yes	No	No	2.25E-06	-0.43	Down
ENSG00000204519	*ZNF551*	Yes	No	No	2.34E-06	0.56	Up
ENSG00000172086	*KRCC1*	Yes	No	No	2.38E-06	-0.66	Down
ENSG00000118181	*RPS25*	Yes	No	No	2.68E-06	-0.50	Down
ENSG00000114923	*SLC4A3*	Yes	No	No	3.35E-06	-0.53	Down
ENSG00000136436	*CALCOCO2*	Yes	No	No	3.40E-06	-0.82	Down
ENSG00000179218	*CALR*	Yes	No	No	3.40E-06	-0.59	Down
ENSG00000105185	*PDCD5*	Yes	No	No	3.50E-06	-0.99	Down
ENSG00000124491	*F13A1*	Yes	No	No	3.50E-06	-1.69	Down
ENSG00000075886	*TUBA3D*	Yes	Yes	Yes	3.66E-06	-1.75	Down
ENSG00000168610	*STAT3*	Yes	No	No	3.81E-06	-1.05	Down
ENSG00000160808	*MYL3*	Yes	No	No	3.84E-06	-1.08	Down
ENSG00000117592	*PRDX6*	Yes	No	No	3.89E-06	-0.89	Down
ENSG00000132522	*GPS2*	Yes	No	No	4.36E-06	-0.34	Down
ENSG00000065054	*SLC9A3R2*	Yes	No	No	4.40E-06	-1.04	Down
ENSG00000118194	*TNNT2*	Yes	No	No	4.40E-06	-0.62	Down
ENSG00000228620	*Z97056.1*	Yes	No	No	4.60E-06	0.89	Up
ENSG00000065978	*YBX1*	Yes	No	No	5.02E-06	-0.55	Down
ENSG00000160051	*IQCC*	Yes	No	No	5.09E-06	0.56	Up
ENSG00000263388	*AC002347.2*	Yes	No	No	5.26E-06	0.51	Up
ENSG00000263843	*AC022211.2*	Yes	No	No	5.48E-06	-0.42	Down
ENSG00000173113	*TRMT112*	Yes	No	No	5.64E-06	-0.31	Down
ENSG00000233110	*AC093797.1*	Yes	No	No	6.01E-06	0.97	Up
ENSG00000089157	*RPLP0*	Yes	No	No	6.43E-06	-1.08	Down
ENSG00000204574	*ABCF1*	Yes	No	No	6.69E-06	-0.47	Down
ENSG00000068976	*PYGM*	Yes	No	Yes	7.34E-06	-1.21	Down
ENSG00000130176	*CNN1*	Yes	Yes	No	7.74E-06	-1.62	Down
ENSG00000175416	*CLTB*	Yes	No	No	8.28E-06	-0.70	Down
ENSG00000198888	*MT-ND1*	Yes	No	No	8.74E-06	-0.64	Down
ENSG00000183255	*PTTG1IP*	Yes	No	No	9.07E-06	-0.50	Down
ENSG00000211445	*GPX3*	Yes	No	No	9.18E-06	-0.83	Down
ENSG00000053524	*MCF2L2*	Yes	No	No	9.66E-06	1.09	Up
ENSG00000262884	*AC015921.1*	Yes	No	No	9.88E-06	-0.88	Down
ENSG00000260465	*AC018557.1*	Yes	No	No	9.89E-06	0.26	Up

ENSG00000161016	*RPL8*	Yes	No	No	9.92E-06	-0.71	Down
ENSG00000007080	*CCDC124*	Yes	No	No	1.10E-05	-0.63	Down
ENSG00000171533	*MAP6*	Yes	No	No	1.18E-05	0.43	Up
ENSG00000286037	*AC011479.4*	Yes	No	No	1.18E-05	0.62	Up
ENSG00000272153	*AL365330.1*	Yes	No	No	1.22E-05	0.30	Up
ENSG00000214530	*STARD10*	Yes	No	Yes	1.23E-05	-1.02	Down
ENSG00000198855	*FICD*	Yes	No	No	1.25E-05	0.50	Up
ENSG00000164694	*FNDC1*	Yes	Yes	No	1.49E-05	2.23	Up
ENSG00000213024	*NUP62*	Yes	No	No	1.50E-05	-0.58	Down
ENSG00000258761	*AC116903.1*	Yes	No	No	1.60E-05	-0.46	Down
ENSG00000063245	*EPN1*	Yes	No	No	1.66E-05	-0.75	Down
ENSG00000071626	*DAZAP1*	Yes	No	No	1.80E-05	-0.80	Down
ENSG00000137312	*FLOT1*	Yes	No	No	1.82E-05	-0.68	Down
ENSG00000187498	*COL4A1*	Yes	No	No	1.84E-05	-0.75	Down
ENSG00000176046	*NUPR1*	Yes	No	No	1.90E-05	-0.79	Down
ENSG00000075624	*ACTB*	Yes	No	No	1.95E-05	-0.98	Down
ENSG00000196557	*CACNA1H*	No	Yes	No	2.00E-05	-1.02	Down
ENSG00000196821	*C6orf106*	Yes	No	No	2.00E-05	-0.59	Down
ENSG00000105953	*OGDH*	Yes	No	No	2.05E-05	-0.68	Down
ENSG00000198959	*TGM2*	Yes	No	No	2.10E-05	-0.96	Down
ENSG00000241135	*LINC00881*	Yes	No	No	2.12E-05	-1.04	Down
ENSG00000089009	*RPL6*	Yes	No	No	2.14E-05	-0.77	Down
ENSG00000188643	*S100A16*	Yes	No	No	2.19E-05	-1.32	Down
ENSG00000126247	*CAPNS1*	Yes	No	No	2.24E-05	-0.69	Down
ENSG00000269886	*AC022382.1*	Yes	No	No	2.26E-05	0.81	Up
ENSG00000160392	*C19orf47*	Yes	No	No	2.42E-05	-0.93	Down
ENSG00000225400	*RAB28P5*	Yes	No	No	2.50E-05	0.48	Up
ENSG00000198467	*TPM2*	Yes	No	No	2.63E-05	-0.80	Down
ENSG00000232767	*AC016825.1*	Yes	No	No	2.64E-05	-0.40	Down
ENSG00000100401	*RANGAP1*	Yes	No	No	2.64E-05	-0.62	Down
ENSG00000136918	*WDR38*	Yes	No	No	2.79E-05	0.37	Up
ENSG00000240583	*AQP1*	Yes	Yes	No	2.82E-05	-1.15	Down
ENSG00000071127	*WDR1*	Yes	No	No	3.01E-05	-0.90	Down
ENSG00000173457	*PPP1R14B*	Yes	No	No	3.01E-05	-0.86	Down
ENSG00000167671	*UBXN6*	Yes	No	No	3.14E-05	-0.37	Down
ENSG00000179943	*FIZ1*	Yes	No	No	3.15E-05	-0.37	Down
ENSG00000199053	*MIR324*	Yes	No	No	3.58E-05	0.67	Up
ENSG00000105974	*CAV1*	Yes	No	No	3.70E-05	-0.72	Down
ENSG00000049768	*FOXP3*	Yes	No	No	3.70E-05	0.49	Up
ENSG00000101421	*CHMP4B*	Yes	No	No	3.75E-05	-0.55	Down

ENSG00000169221	*TBC1D10B*	Yes	No	No	4.23E-05	-0.37	Down
ENSG00000073008	*PVR*	Yes	No	No	4.27E-05	-1.04	Down
ENSG00000125651	*GTF2F1*	Yes	No	No	4.51E-05	-0.49	Down
ENSG00000170142	*UBE2E1*	Yes	No	No	4.54E-05	-0.30	Down
ENSG00000182054	*IDH2*	Yes	No	No	5.07E-05	-0.86	Down
ENSG00000120049	*KCNIP2*	Yes	No	No	5.08E-05	-0.99	Down
ENSG00000131459	*GFPT2*	Yes	No	Yes	5.09E-05	-1.80	Down
ENSG00000229273	*BX664615.1*	Yes	No	No	5.50E-05	0.49	Up
ENSG00000269139	*AC010336.5*	Yes	No	No	5.50E-05	-0.67	Down
ENSG00000232803	*SLCO4A1-AS1*	No	Yes	Yes	5.72E-05	-1.54	Down
ENSG00000188163	*FAM166A*	Yes	No	No	5.87E-05	-0.96	Down
ENSG00000012211	*PRICKLE3*	Yes	No	No	6.21E-05	-0.62	Down
ENSG00000175166	*PSMD2*	Yes	No	No	6.73E-05	-0.42	Down
ENSG00000114854	*TNNC1*	Yes	No	No	7.34E-05	-0.57	Down
ENSG00000177731	*FLII*	Yes	No	No	7.39E-05	-0.57	Down
ENSG00000130816	*DNMT1*	Yes	No	No	7.56E-05	-0.87	Down
ENSG00000187984	*ANKRD19P*	Yes	No	No	7.64E-05	0.44	Up
ENSG00000258232	*AC125611.3*	Yes	No	No	7.73E-05	-0.80	Down
ENSG00000081803	*CADPS2*	No	No	Yes	7.82E-05	-1.39	Down
ENSG00000116350	*SRSF4*	Yes	No	No	8.51E-05	-0.25	Down
ENSG00000185475	*TMEM179B*	Yes	No	No	8.71E-05	-0.45	Down
ENSG00000196549	*MME*	No	Yes	Yes	8.75E-05	1.96	Up
ENSG00000039319	*ZFYVE16*	Yes	No	No	8.81E-05	0.68	Up
ENSG00000170417	*TMEM182*	Yes	No	No	8.95E-05	1.29	Up
ENSG00000279033	*AC090984.1*	Yes	No	No	8.99E-05	0.31	Up
ENSG00000083845	*RPS5*	Yes	No	No	9.42E-05	-0.70	Down
ENSG00000101439	*CST3*	Yes	No	No	9.42E-05	-0.63	Down
ENSG00000198874	*TYW1*	Yes	No	No	9.42E-05	0.28	Up
ENSG00000213694	*S1PR3*	Yes	No	Yes	9.42E-05	-1.26	Down
ENSG00000188227	*ZNF793*	Yes	No	No	9.86E-05	0.58	Up
ENSG00000163468	*CCT3*	Yes	No	No	1.02E-04	-0.85	Down
ENSG00000213442	*RPL18AP3*	Yes	No	No	1.06E-04	-0.85	Down
ENSG00000163431	*LMOD1*	No	No	Yes	1.07E-04	-1.02	Down
ENSG00000066230	*SLC9A3*	Yes	Yes	No	1.08E-04	1.27	Up
ENSG00000136875	*PRPF4*	Yes	No	No	1.08E-04	-0.35	Down
ENSG00000279314	*AC002525.1*	Yes	No	No	1.08E-04	0.64	Up
ENSG00000104848	*KCNA7*	Yes	No	Yes	1.13E-04	-1.66	Down
ENSG00000235958	*UBOX5-AS1*	Yes	No	No	1.16E-04	0.60	Up
ENSG00000106631	*MYL7*	Yes	No	No	1.17E-04	-1.23	Down
ENSG00000139329	*LUM*	No	Yes	Yes	1.17E-04	1.26	Up

ENSG00000080709	*KCNN2*	Yes	No	Yes	1.19E-04	-1.44	Down
ENSG00000159403	*C1R*	Yes	No	No	1.19E-04	-1.02	Down
ENSG00000065518	*NDUFB4*	Yes	No	No	1.21E-04	-0.80	Down
ENSG00000209082	*MT-TL1*	Yes	No	No	1.23E-04	-1.05	Down
ENSG00000167680	*SEMA6B*	Yes	No	No	1.24E-04	-1.39	Down
ENSG00000014216	*CAPN1*	Yes	No	No	1.25E-04	-0.47	Down
ENSG00000159189	*C1QC*	Yes	No	No	1.29E-04	-1.15	Down
ENSG00000186714	*CCDC73*	Yes	No	No	1.31E-04	-0.44	Down
ENSG00000101144	*BMP7*	Yes	No	No	1.36E-04	-1.81	Down
ENSG00000036257	*CUL3*	Yes	No	No	1.38E-04	-0.66	Down
ENSG00000143870	*PDIA6*	Yes	No	No	1.38E-04	-0.61	Down
ENSG00000249825	*AC012636.1*	Yes	No	No	1.45E-04	0.99	Up
ENSG00000187147	*RNF220*	Yes	No	No	1.52E-04	-0.48	Down
ENSG00000285577	*AC019127.1*	Yes	No	No	1.54E-04	0.76	Up
ENSG00000116260	*QSOX1*	Yes	No	No	1.61E-04	-1.20	Down
ENSG00000134765	*DSC1*	No	Yes	No	1.68E-04	1.51	Up
ENSG00000178764	*ZHX2*	Yes	No	No	1.74E-04	-0.60	Down
ENSG00000198034	*RPS4X*	Yes	No	No	1.75E-04	-0.62	Down
ENSG00000132475	*H3F3B*	Yes	No	No	1.79E-04	-0.49	Down
ENSG00000141965	*FEM1A*	Yes	No	No	1.84E-04	-0.77	Down
ENSG00000233138	*AL023584.1*	Yes	No	No	1.85E-04	0.74	Up
ENSG00000138326	*RPS24*	Yes	No	No	1.85E-04	-0.72	Down
ENSG00000165124	*SVEP1*	Yes	No	Yes	1.88E-04	0.99	Up
ENSG00000112306	*RPS12*	Yes	No	No	1.91E-04	-0.79	Down
ENSG00000160447	*PKN3*	Yes	No	No	1.93E-04	-0.87	Down
ENSG00000101347	*SAMHD1*	Yes	No	No	1.94E-04	-1.04	Down
ENSG00000277801	*AL138478.1*	Yes	No	No	1.96E-04	-0.32	Down
ENSG00000054654	*SYNE2*	Yes	No	No	1.98E-04	-0.71	Down
ENSG00000166123	*GPT2*	No	No	Yes	2.01E-04	-1.21	Down
ENSG00000114554	*PLXNA1*	Yes	No	No	2.12E-04	-1.03	Down
ENSG00000142330	*CAPN10*	Yes	No	No	2.13E-04	-0.68	Down
ENSG00000167995	*BEST1*	Yes	No	No	2.21E-04	-0.46	Down
ENSG00000103056	*SMPD3*	Yes	No	No	2.24E-04	0.41	Up
ENSG00000225098	*BCRP1*	Yes	No	No	2.26E-04	-1.71	Down
ENSG00000204673	*AKT1S1*	Yes	No	No	2.26E-04	-0.40	Down
ENSG00000135423	*GLS2*	Yes	No	No	2.26E-04	0.56	Up
ENSG00000113456	*RAD1*	Yes	No	No	2.26E-04	-0.64	Down
ENSG00000113721	*PDGFRB*	Yes	No	No	2.26E-04	-0.97	Down
ENSG00000286077	*AL158141.1*	Yes	No	No	2.28E-04	-0.65	Down
ENSG00000106336	*FBXO24*	Yes	No	No	2.34E-04	1.11	Up

ENSG00000226009	*KCNIP2-AS1*	Yes	No	No	2.34E-04	-1.03	Down
ENSG00000244945	*AC136604.2*	Yes	No	No	2.36E-04	-0.32	Down
ENSG00000149428	*HYOU1*	Yes	No	No	2.42E-04	-0.87	Down
ENSG00000092969	*TGFB2*	Yes	No	No	2.51E-04	1.13	Up
ENSG00000264578	*AC009630.3*	Yes	No	No	2.65E-04	-0.27	Down
ENSG00000254473	*AL354920.1*	Yes	No	No	2.65E-04	0.56	Up
ENSG00000270112	*AC090241.2*	Yes	No	No	2.71E-04	0.88	Up
ENSG00000127616	*SMARCA4*	Yes	No	No	2.75E-04	-0.51	Down
ENSG00000011295	*TTC19*	Yes	No	No	2.77E-04	-0.32	Down
ENSG00000214783	*POLR2J4*	Yes	No	No	2.80E-04	-0.41	Down
ENSG00000159251	*ACTC1*	Yes	No	Yes	2.85E-04	-0.75	Down
ENSG00000105618	*PRPF31*	Yes	No	No	3.06E-04	-0.38	Down
ENSG00000267470	*ZNF571-AS1*	Yes	No	No	3.19E-04	0.91	Up
ENSG00000143740	*SNAP47*	Yes	No	No	3.24E-04	0.45	Up
ENSG00000197756	*RPL37A*	Yes	No	No	3.29E-04	-0.59	Down
ENSG00000234617	*SNRK-AS1*	Yes	No	No	3.49E-04	0.72	Up
ENSG00000088992	*TESC*	No	No	Yes	3.50E-04	-0.66	Down
ENSG00000007516	*BAIAP3*	Yes	No	No	3.52E-04	-0.41	Down
ENSG00000225670	*CADM3-AS1*	Yes	No	Yes	3.52E-04	-1.09	Down
ENSG00000279159	*AC003681.1*	Yes	No	No	3.54E-04	0.31	Up
ENSG00000264112	*AC015813.1*	Yes	No	No	3.55E-04	1.13	Up
ENSG00000226143	*Z98752.1*	Yes	No	No	3.60E-04	0.62	Up
ENSG00000163359	*COL6A3*	Yes	No	No	3.76E-04	-0.82	Down
ENSG00000064601	*CTSA*	Yes	No	No	3.88E-04	-0.41	Down
ENSG00000227199	*ST7-AS1*	Yes	No	No	3.90E-04	-0.54	Down
ENSG00000116871	*MAP7D1*	Yes	No	No	3.95E-04	-0.44	Down
ENSG00000272750	*AL592148.3*	Yes	No	No	3.96E-04	-0.16	Down
ENSG00000117394	*SLC2A1*	Yes	No	No	4.07E-04	-0.78	Down
ENSG00000076344	*RGS11*	Yes	No	No	4.21E-04	0.83	Up
ENSG00000008311	*AASS*	Yes	No	No	4.27E-04	-0.92	Down
ENSG00000261065	*AL592146.1*	Yes	No	No	4.41E-04	-0.79	Down
ENSG00000103415	*HMOX2*	Yes	No	No	4.46E-04	-0.61	Down
ENSG00000168268	*NT5DC2*	No	No	Yes	4.70E-04	-1.08	Down
ENSG00000108592	*FTSJ3*	Yes	No	No	4.74E-04	-0.34	Down
ENSG00000177600	*RPLP2*	Yes	No	No	5.08E-04	-0.75	Down
ENSG00000102466	*FGF14*	Yes	No	No	5.27E-04	1.24	Up
ENSG00000106484	*MEST*	Yes	No	No	5.37E-04	0.80	Up
ENSG00000018280	*SLC11A1*	No	Yes	No	5.53E-04	-1.31	Down
ENSG00000132589	*FLOT2*	Yes	No	No	5.55E-04	-0.94	Down
ENSG00000175463	*TBC1D10C*	Yes	No	No	5.55E-04	0.93	Up

ENSG00000179604	*CDC42EP4*	Yes	No	No	5.58E-04	-1.11	Down
ENSG00000271976	*AC012467.2*	Yes	No	No	5.64E-04	-0.94	Down
ENSG00000090061	*CCNK*	Yes	No	No	5.78E-04	-0.28	Down
ENSG00000140199	*SLC12A6*	Yes	No	No	5.90E-04	-0.23	Down
ENSG00000187187	*ZNF546*	Yes	No	No	5.91E-04	0.56	Up
ENSG00000135974	*C2orf49*	Yes	No	No	6.00E-04	0.85	Up
ENSG00000104904	*OAZ1*	Yes	No	No	6.11E-04	-0.77	Down
ENSG00000129116	*PALLD*	Yes	No	No	6.23E-04	-0.57	Down
ENSG00000148229	*POLE3*	Yes	No	No	6.27E-04	-0.66	Down
ENSG00000134871	*COL4A2*	Yes	No	No	6.49E-04	-0.58	Down
ENSG00000135720	*DYNC1LI2*	Yes	No	No	6.69E-04	0.75	Up
ENSG00000129255	*MPDU1*	Yes	No	No	6.71E-04	-0.37	Down
ENSG00000116962	*NID1*	Yes	Yes	No	6.94E-04	-0.98	Down
ENSG00000134030	*CTIF*	Yes	No	No	7.14E-04	-0.53	Down
ENSG00000173641	*HSPB7*	Yes	No	No	7.23E-04	-0.57	Down
ENSG00000273151	*AC073957.3*	Yes	No	No	7.40E-04	0.54	Up
ENSG00000267128	*RNF157-AS1*	Yes	No	No	7.48E-04	-0.94	Down
ENSG00000159335	*PTMS*	Yes	No	No	7.88E-04	-0.76	Down
ENSG00000155115	*GTF3C6*	Yes	No	No	7.89E-04	-0.70	Down
ENSG00000100968	*NFATC4*	Yes	Yes	No	7.93E-04	-1.20	Down
ENSG00000163082	*SGPP2*	No	Yes	No	8.02E-04	-1.46	Down
ENSG00000128591	*FLNC*	Yes	No	No	8.13E-04	-0.88	Down
ENSG00000140564	*FURIN*	Yes	No	No	8.14E-04	-0.81	Down
ENSG00000143321	*HDGF*	Yes	No	No	8.22E-04	-0.54	Down
ENSG00000142459	*EVI5L*	Yes	No	No	8.43E-04	-0.90	Down
ENSG00000110697	*PITPNM1*	Yes	No	No	8.50E-04	-0.70	Down
ENSG00000132716	*DCAF8*	Yes	No	No	8.57E-04	0.44	Up
ENSG00000229780	*UBE2Q1-AS1*	Yes	No	No	8.71E-04	-0.48	Down
ENSG00000118292	*C1orf54*	Yes	No	No	8.74E-04	0.34	Up
ENSG00000145494	*NDUFS6*	Yes	No	No	8.78E-04	-0.82	Down
ENSG00000230927	*TMBIM7P*	Yes	No	No	8.87E-04	0.65	Up
ENSG00000132507	*EIF5A*	Yes	No	No	9.14E-04	-0.48	Down
ENSG00000261684	*AC018362.1*	Yes	No	No	9.26E-04	0.25	Up
ENSG00000166831	*RBPMS2*	Yes	No	No	9.33E-04	-0.89	Down
ENSG00000237686	*AL109615.3*	Yes	No	No	9.52E-04	0.80	Up
ENSG00000115593	*SMYD1*	Yes	No	No	9.53E-04	-0.85	Down
ENSG00000185739	*SRL*	Yes	No	No	1.06E-03	-0.60	Down
ENSG00000113758	*DBN1*	No	No	Yes	1.08E-03	-0.87	Down
ENSG00000164879	*CA3*	No	No	Yes	1.09E-03	1.42	Up
ENSG00000181577	*C6orf223*	Yes	No	No	1.10E-03	1.10	Up

ENSG00000136942	*RPL35*	Yes	No	No	1.10E-03	-0.58	Down
ENSG00000142910	*TINAGL1*	Yes	No	No	1.10E-03	-0.66	Down
ENSG00000006007	*GDE1*	Yes	No	No	1.11E-03	-0.74	Down
ENSG00000163638	*ADAMTS9*	No	No	Yes	1.14E-03	-1.20	Down
ENSG00000179262	*RAD23A*	Yes	No	No	1.14E-03	-0.43	Down
ENSG00000173372	*C1QA*	Yes	No	No	1.21E-03	-0.99	Down
ENSG00000186350	*RXRA*	Yes	No	No	1.21E-03	-0.68	Down
ENSG00000065882	*TBC1D1*	Yes	No	No	1.23E-03	-0.83	Down
ENSG00000121440	*PDZRN3*	Yes	No	No	1.23E-03	-0.45	Down
ENSG00000272540	*AL662797.1*	Yes	No	No	1.25E-03	-0.58	Down
ENSG00000196437	*ZNF569*	Yes	No	No	1.27E-03	0.66	Up
ENSG00000107263	*RAPGEF1*	Yes	No	No	1.28E-03	-0.66	Down
ENSG00000185222	*TCEAL9*	Yes	No	No	1.32E-03	-0.52	Down
ENSG00000151729	*SLC25A4*	Yes	No	No	1.40E-03	-0.54	Down
ENSG00000158859	*ADAMTS4*	No	No	No	1.44E-03	-1.15	Down
ENSG00000185896	*LAMP1*	Yes	No	No	1.44E-03	-0.64	Down
ENSG00000261276	*AP003071.3*	Yes	No	No	1.47E-03	-1.06	Down
ENSG00000238045	*AC009133.1*	Yes	No	No	1.48E-03	-0.45	Down
ENSG00000188677	*PARVB*	Yes	No	No	1.49E-03	-0.81	Down
ENSG00000237732	*AC010980.1*	Yes	No	No	1.50E-03	-1.20	Down
ENSG00000131653	*TRAF7*	Yes	No	No	1.50E-03	-0.62	Down
ENSG00000168140	*VASN*	Yes	Yes	No	1.51E-03	-0.89	Down
ENSG00000115705	*TPO*	No	Yes	No	1.52E-03	-1.15	Down
ENSG00000188467	*SLC24A5*	Yes	No	No	1.53E-03	0.48	Up
ENSG00000130508	*PXDN*	Yes	No	No	1.53E-03	-1.11	Down
ENSG00000069122	*ADGRF5*	Yes	No	No	1.55E-03	-0.60	Down
ENSG00000239857	*GET4*	Yes	No	No	1.56E-03	0.73	Up
ENSG00000168256	*NKIRAS2*	Yes	No	No	1.56E-03	-0.37	Down
ENSG00000135519	*KCNH3*	Yes	No	No	1.57E-03	-0.63	Down
ENSG00000124562	*SNRPC*	Yes	No	No	1.59E-03	-0.82	Down
ENSG00000196182	*STK40*	Yes	No	No	1.62E-03	-0.71	Down
ENSG00000131652	*THOC6*	Yes	No	No	1.62E-03	-0.58	Down
ENSG00000282917	*AC107068.2*	No	No	Yes	1.63E-03	-1.40	Down
ENSG00000179632	*MAF1*	Yes	No	No	1.66E-03	-0.47	Down
ENSG00000165655	*ZNF503*	Yes	No	No	1.68E-03	-0.99	Down
ENSG00000102225	*CDK16*	Yes	No	No	1.73E-03	-0.70	Down
ENSG00000106483	*SFRP4*	Yes	Yes	No	1.78E-03	1.52	Up
ENSG00000104907	*TRMT1*	Yes	No	No	1.83E-03	-0.71	Down
ENSG00000231437	*LINC01750*	Yes	No	No	1.83E-03	-0.89	Down
ENSG00000153902	*LGI4*	Yes	No	No	1.84E-03	-0.76	Down

ENSG00000232907	*DLGAP4-AS1*	Yes	No	No	1.87E-03	0.29	Up
ENSG00000022840	*RNF10*	Yes	No	No	1.90E-03	-0.51	Down
ENSG00000064300	*NGFR*	No	Yes	No	1.90E-03	-1.28	Down
ENSG00000179636	*TPPP2*	Yes	No	No	1.91E-03	-0.50	Down
ENSG00000198892	*SHISA4*	Yes	No	No	1.91E-03	-1.07	Down
ENSG00000143545	*RAB13*	Yes	No	No	1.93E-03	-0.67	Down
ENSG00000132000	*PODNL1*	Yes	No	No	1.94E-03	1.41	Up
ENSG00000138031	*ADCY3*	Yes	No	No	2.03E-03	-0.77	Down
ENSG00000184009	*ACTG1*	Yes	No	No	2.07E-03	-0.73	Down
ENSG00000140443	*IGF1R*	Yes	No	No	2.07E-03	0.62	Up
ENSG00000250159	*AC106791.1*	Yes	No	No	2.07E-03	-0.79	Down
ENSG00000115241	*PPM1G*	Yes	No	No	2.09E-03	-0.57	Down
ENSG00000269881	*AC004754.1*	Yes	No	No	2.10E-03	0.58	Up
ENSG00000164733	*CTSB*	Yes	No	No	2.10E-03	-0.50	Down
ENSG00000171476	*HOPX*	No	No	Yes	2.13E-03	-1.47	Down
ENSG00000141568	*FOXK2*	Yes	No	No	2.13E-03	-0.35	Down
ENSG00000126934	*MAP2K2*	Yes	No	No	2.13E-03	-0.83	Down
ENSG00000259352	*AC091117.1*	Yes	No	No	2.24E-03	-0.48	Down
ENSG00000259031	*AL845552.2*	Yes	No	No	2.25E-03	1.36	Up
ENSG00000197912	*SPG7*	Yes	No	No	2.39E-03	-0.44	Down
ENSG00000162706	*CADM3*	No	No	Yes	2.41E-03	-1.26	Down
ENSG00000110400	*NECTIN1*	Yes	Yes	No	2.60E-03	-1.03	Down
ENSG00000156453	*PCDH1*	Yes	No	No	2.64E-03	-0.97	Down
ENSG00000173114	*LRRN3*	No	No	Yes	2.65E-03	-1.57	Down
ENSG00000267255	*AC011498.3*	Yes	No	No	2.65E-03	-0.84	Down
ENSG00000198522	*GPN1*	Yes	No	No	2.67E-03	-0.19	Down
ENSG00000161647	*MPP3*	Yes	No	No	2.68E-03	-1.09	Down
ENSG00000241158	*ADAMTS9-AS1*	Yes	No	Yes	2.72E-03	-1.15	Down
ENSG00000233223	*AC016876.1*	Yes	No	No	2.72E-03	-0.61	Down
ENSG00000149657	*LSM14B*	Yes	No	No	2.76E-03	-0.64	Down
ENSG00000117281	*CD160*	Yes	No	No	2.86E-03	0.45	Up
ENSG00000010327	*STAB1*	No	Yes	No	2.88E-03	-0.62	Down
ENSG00000156885	*COX6A2*	Yes	No	No	2.97E-03	-0.59	Down
ENSG00000224597	*SVIL-AS1*	Yes	No	No	3.03E-03	0.31	Up
ENSG00000180354	*MTURN*	No	No	Yes	3.03E-03	0.96	Up
ENSG00000140987	*ZSCAN32*	Yes	No	No	3.04E-03	0.30	Up
ENSG00000175832	*ETV4*	No	No	Yes	3.06E-03	1.28	Up
ENSG00000142494	*SLC47A1*	Yes	No	No	3.07E-03	1.04	Up
ENSG00000113504	*SLC12A7*	Yes	No	No	3.08E-03	-0.39	Down
ENSG00000100243	*CYB5R3*	Yes	No	No	3.16E-03	-0.56	Down

ENSG00000171853	*TRAPPC12*	Yes	No	No	3.19E-03	-0.50	Down
ENSG00000158315	*RHBDL2*	Yes	No	No	3.23E-03	0.79	Up
ENSG00000166313	*APBB1*	Yes	No	No	3.23E-03	-0.63	Down
ENSG00000088256	*GNA11*	Yes	No	No	3.23E-03	-0.57	Down
ENSG00000011347	*SYT7*	Yes	No	No	3.30E-03	-0.96	Down
ENSG00000165175	*MID1IP1*	Yes	No	No	3.47E-03	-1.00	Down
ENSG00000265287	*AC005726.5*	Yes	No	No	3.55E-03	0.51	Up
ENSG00000168309	*FAM107A*	Yes	No	No	3.55E-03	-0.97	Down
ENSG00000117395	*EBNA1BP2*	Yes	No	No	3.57E-03	-0.78	Down
ENSG00000078098	*FAP*	No	No	Yes	3.57E-03	1.05	Up
ENSG00000011304	*PTBP1*	Yes	No	No	3.59E-03	-0.93	Down
ENSG00000078808	*SDF4*	Yes	No	No	3.61E-03	-0.54	Down
ENSG00000249669	*CARMN*	No	No	Yes	3.61E-03	-0.82	Down
ENSG00000174903	*RAB1B*	Yes	No	No	3.64E-03	-0.67	Down
ENSG00000185624	*P4HB*	Yes	No	No	3.65E-03	-0.52	Down
ENSG00000187189	*TSPYL4*	Yes	No	No	3.72E-03	-0.58	Down
ENSG00000128272	*ATF4*	Yes	No	No	3.74E-03	-0.77	Down
ENSG00000205189	*ZBTB10*	Yes	No	No	3.74E-03	0.50	Up
ENSG00000129351	*ILF3*	Yes	No	No	3.77E-03	-0.46	Down
ENSG00000023902	*PLEKHO1*	Yes	No	No	3.80E-03	-0.90	Down
ENSG00000100106	*TRIOBP*	Yes	No	No	3.83E-03	-0.51	Down
ENSG00000109107	*ALDOC*	Yes	No	No	3.84E-03	-0.82	Down
ENSG00000123144	*TRIR*	Yes	No	No	3.84E-03	-0.56	Down
ENSG00000258260	*AC073896.5*	Yes	No	No	3.97E-03	-0.42	Down
ENSG00000100296	*THOC5*	Yes	No	No	3.98E-03	-0.87	Down
ENSG00000162817	*C1orf115*	Yes	No	No	3.98E-03	-1.03	Down
ENSG00000165672	*PRDX3*	Yes	No	No	3.99E-03	-0.74	Down
ENSG00000249087	*ZNF436-AS1*	No	No	Yes	4.01E-03	0.90	Up
ENSG00000136848	*DAB2IP*	Yes	No	No	4.06E-03	-0.55	Down
ENSG00000097046	*CDC7*	Yes	No	No	4.10E-03	-1.29	Down
ENSG00000124795	*DEK*	Yes	No	No	4.17E-03	-0.56	Down
ENSG00000174038	*C9orf131*	Yes	No	No	4.17E-03	0.72	Up
ENSG00000166106	*ADAMTS15*	No	No	Yes	4.17E-03	-0.90	Down
ENSG00000274211	*SOCS7*	Yes	No	No	4.18E-03	0.81	Up
ENSG00000122176	*FMOD*	No	No	Yes	4.27E-03	1.12	Up
ENSG00000064225	*ST3GAL6*	Yes	No	No	4.32E-03	0.50	Up
ENSG00000151883	*PARP8*	Yes	No	No	4.38E-03	0.70	Up
ENSG00000113916	*BCL6*	Yes	No	No	4.48E-03	-0.85	Down
ENSG00000140553	*UNC45A*	Yes	No	No	4.49E-03	-0.21	Down
ENSG00000210100	*MT-TI*	Yes	No	No	4.68E-03	-0.66	Down

ENSG00000091527	*CDV3*	Yes	No	No	4.71E-03	-0.44	Down
ENSG00000275457	*AL117332.1*	Yes	No	No	4.88E-03	-0.36	Down
ENSG00000148484	*RSU1*	Yes	No	No	5.03E-03	-0.79	Down
ENSG00000034533	*ASTE1*	Yes	No	No	5.04E-03	0.18	Up
ENSG00000230532	*AC091133.1*	Yes	No	No	5.30E-03	-0.36	Down
ENSG00000142227	*EMP3*	Yes	No	No	5.45E-03	-0.85	Down
ENSG00000127585	*FBXL16*	No	No	Yes	5.48E-03	1.60	Up
ENSG00000152082	*MZT2B*	Yes	No	No	5.54E-03	-0.52	Down
ENSG00000122033	*MTIF3*	Yes	No	No	5.57E-03	-0.42	Down
ENSG00000174640	*SLCO2A1*	No	Yes	No	5.60E-03	-1.06	Down
ENSG00000197043	*ANXA6*	Yes	No	No	5.62E-03	-0.62	Down
ENSG00000146147	*MLIP*	Yes	No	No	5.73E-03	-0.72	Down
ENSG00000165487	*MICU2*	Yes	No	No	5.78E-03	-0.88	Down
ENSG00000090013	*BLVRB*	Yes	No	No	5.86E-03	-0.83	Down
ENSG00000079999	*KEAP1*	Yes	No	No	5.88E-03	-0.76	Down
ENSG00000257225	*AC079601.1*	Yes	No	Yes	5.99E-03	0.71	Up
ENSG00000182481	*KPNA2*	Yes	No	No	6.01E-03	-1.01	Down
ENSG00000268442	*AC073534.1*	Yes	No	No	6.01E-03	1.00	Up
ENSG00000124380	*SNRNP27*	Yes	No	No	6.02E-03	-0.72	Down
ENSG00000167723	*TRPV3*	No	No	Yes	6.02E-03	1.28	Up
ENSG00000228113	*AC003991.1*	Yes	No	No	6.02E-03	-0.61	Down
ENSG00000137959	*IFI44L*	No	No	Yes	6.03E-03	1.14	Up
ENSG00000067829	*IDH3G*	Yes	No	No	6.05E-03	-0.49	Down
ENSG00000068001	*HYAL2*	Yes	No	No	6.14E-03	-1.08	Down
ENSG00000232973	*CYP1B1-AS1*	Yes	No	No	6.14E-03	0.90	Up
ENSG00000168079	*SCARA5*	Yes	No	No	6.26E-03	-1.15	Down
ENSG00000175183	*CSRP2*	No	No	Yes	6.26E-03	-0.92	Down
ENSG00000210117	*MT-TW*	Yes	No	No	6.26E-03	-0.98	Down
ENSG00000022267	*FHL1*	Yes	No	No	6.29E-03	-0.67	Down
ENSG00000160685	*ZBTB7B*	Yes	No	No	6.40E-03	-0.79	Down
ENSG00000185201	*IFITM2*	No	No	No	6.57E-03	-1.03	Down
ENSG00000122378	*PRXL2A*	Yes	No	No	6.59E-03	-0.75	Down
ENSG00000222019	*URAHP*	Yes	No	No	6.68E-03	0.31	Up
ENSG00000109339	*MAPK10*	Yes	No	No	6.68E-03	0.34	Up
ENSG00000164236	*ANKRD33B*	No	Yes	No	6.69E-03	0.97	Up
ENSG00000134046	*MBD2*	Yes	No	No	6.78E-03	-0.80	Down
ENSG00000278367	*AL356652.1*	Yes	No	No	6.98E-03	-0.77	Down
ENSG00000011243	*AKAP8L*	Yes	No	No	7.18E-03	-0.45	Down
ENSG00000166794	*PPIB*	Yes	No	No	7.26E-03	-0.68	Down
ENSG00000257553	*AC034102.4*	Yes	No	No	7.28E-03	-0.31	Down

ENSG00000157653	*C9orf43*	Yes	No	No	7.33E-03	-0.42	Down
ENSG00000166676	*TVP23A*	Yes	No	No	7.37E-03	-0.22	Down
ENSG00000138080	*EMILIN1*	Yes	No	No	7.47E-03	-0.74	Down
ENSG00000099875	*MKNK2*	Yes	No	No	7.57E-03	-0.76	Down
ENSG00000278616	*BEND3P3*	Yes	No	No	7.59E-03	0.90	Up
ENSG00000164897	*TMUB1*	Yes	No	No	7.72E-03	-0.69	Down
ENSG00000173264	*GPR137*	Yes	No	No	7.79E-03	-0.35	Down
ENSG00000204463	*BAG6*	Yes	No	No	7.79E-03	-0.38	Down
ENSG00000161203	*AP2M1*	Yes	No	No	7.91E-03	-0.48	Down
ENSG00000164253	*WDR41*	Yes	No	No	7.91E-03	0.57	Up
ENSG00000100345	*MYH9*	Yes	No	No	8.11E-03	-0.48	Down
ENSG00000162551	*ALPL*	No	No	No	8.13E-03	-1.28	Down
ENSG00000075413	*MARK3*	Yes	No	No	8.31E-03	-0.72	Down
ENSG00000213889	*PPM1N*	Yes	No	No	8.34E-03	-0.43	Down
ENSG00000130725	*UBE2M*	Yes	No	No	8.34E-03	-0.53	Down
ENSG00000110925	*CSRNP2*	Yes	No	No	8.36E-03	0.33	Up
ENSG00000255422	*AP002954.1*	Yes	No	No	8.46E-03	0.59	Up
ENSG00000171552	*BCL2L1*	Yes	No	No	8.54E-03	-0.90	Down
ENSG00000116176	*TPSG1*	No	Yes	No	8.58E-03	-2.22	Down
ENSG00000179564	*LSMEM2*	Yes	No	No	8.66E-03	-0.37	Down
ENSG00000197586	*ENTPD6*	Yes	No	No	8.81E-03	-0.66	Down
ENSG00000151365	*THRSP*	No	No	No	8.81E-03	1.71	Up
ENSG00000142733	*MAP3K6*	No	No	No	8.95E-03	-0.91	Down
ENSG00000274386	*TMEM269*	Yes	No	No	9.18E-03	-0.50	Down
ENSG00000166173	*LARP6*	Yes	No	No	9.45E-03	-0.69	Down
ENSG00000064666	*CNN2*	Yes	No	No	9.51E-03	-0.92	Down
ENSG00000141198	*TOM1L1*	Yes	No	No	9.52E-03	0.31	Up
ENSG00000140941	*MAP1LC3B*	Yes	No	No	9.54E-03	-0.36	Down
ENSG00000101224	*CDC25B*	No	Yes	No	9.58E-03	-0.87	Down
ENSG00000155090	*KLF10*	Yes	No	No	9.58E-03	-1.19	Down
ENSG00000128309	*MPST*	Yes	No	No	9.75E-03	-0.94	Down
ENSG00000205726	*ITSN1*	Yes	No	No	9.78E-03	-0.35	Down
ENSG00000267064	*UXT-AS1*	Yes	No	No	9.91E-03	-0.38	Down
ENSG00000119979	*FAM45A*	Yes	No	No	1.00E-02	0.47	Up
ENSG00000187840	*EIF4EBP1*	Yes	No	No	1.00E-02	-1.16	Down
ENSG00000162878	*PKDCC*	Yes	No	No	1.03E-02	-0.78	Down
ENSG00000123143	*PKN1*	Yes	No	No	1.03E-02	-0.70	Down
ENSG00000158246	*TENT5B*	No	No	No	1.03E-02	-1.35	Down
ENSG00000170734	*POLH*	Yes	No	No	1.06E-02	0.70	Up
ENSG00000185033	*SEMA4B*	No	Yes	No	1.06E-02	-0.88	Down

ENSG00000115484	*CCT4*	Yes	No	No	1.06E-02	-0.61	Down
ENSG00000072274	*TFRC*	Yes	No	No	1.07E-02	-0.99	Down
ENSG00000145476	*CYP4V2*	Yes	No	No	1.08E-02	1.11	Up
ENSG00000135424	*ITGA7*	Yes	No	No	1.08E-02	-0.34	Down
ENSG00000105894	*PTN*	No	Yes	No	1.09E-02	0.84	Up
ENSG00000170889	*RPS9*	Yes	No	No	1.09E-02	-0.54	Down
ENSG00000235902	*AC108472.1*	No	No	Yes	1.09E-02	1.19	Up
ENSG00000102103	*PQBP1*	Yes	No	No	1.09E-02	-0.44	Down
ENSG00000160886	*LY6K*	Yes	No	No	1.12E-02	1.57	Up
ENSG00000106290	*TAF6*	Yes	No	No	1.13E-02	-0.23	Down
ENSG00000108679	*LGALS3BP*	Yes	No	No	1.13E-02	-0.72	Down
ENSG00000170584	*NUDCD2*	Yes	No	No	1.13E-02	-0.41	Down
ENSG00000172059	*KLF11*	Yes	No	No	1.13E-02	0.57	Up
ENSG00000135049	*AGTPBP1*	Yes	No	No	1.14E-02	-0.98	Down
ENSG00000153823	*PID1*	Yes	No	No	1.14E-02	-1.18	Down
ENSG00000186184	*POLR1D*	Yes	No	No	1.15E-02	-0.56	Down
ENSG00000113296	*THBS4*	Yes	No	No	1.18E-02	0.79	Up
ENSG00000169564	*PCBP1*	Yes	No	No	1.19E-02	-0.50	Down
ENSG00000183067	*IGSF5*	Yes	No	No	1.20E-02	-0.96	Down
ENSG00000160539	*PLPP7*	Yes	No	No	1.21E-02	-0.60	Down
ENSG00000087274	*ADD1*	Yes	No	No	1.21E-02	-0.49	Down
ENSG00000154928	*EPHB1*	Yes	No	No	1.22E-02	-0.89	Down
ENSG00000104812	*GYS1*	Yes	No	No	1.24E-02	-0.48	Down
ENSG00000185483	*ROR1*	No	No	Yes	1.24E-02	0.85	Up
ENSG00000177706	*FAM20C*	Yes	No	No	1.29E-02	-0.95	Down
ENSG00000234638	*AC053503.4*	Yes	No	No	1.30E-02	-0.36	Down
ENSG00000198355	*PIM3*	Yes	No	No	1.30E-02	-0.88	Down
ENSG00000231500	*RPS18*	Yes	No	No	1.33E-02	-0.39	Down
ENSG00000100234	*TIMP3*	No	Yes	No	1.33E-02	-0.62	Down
ENSG00000234745	*HLA-B*	Yes	No	No	1.36E-02	-0.48	Down
ENSG00000136770	*DNAJC1*	Yes	No	No	1.37E-02	-0.81	Down
ENSG00000185924	*RTN4RL1*	No	No	Yes	1.37E-02	-1.17	Down
ENSG00000142748	*FCN3*	No	No	Yes	1.38E-02	-1.57	Down
ENSG00000043591	*ADRB1*	Yes	No	No	1.38E-02	-0.90	Down
ENSG00000087495	*PHACTR3*	No	No	No	1.38E-02	-1.57	Down
ENSG00000273212	*AC000068.2*	Yes	No	No	1.39E-02	-0.40	Down
ENSG00000231389	*HLA-DPA1*	No	No	Yes	1.40E-02	0.58	Up
ENSG00000178105	*DDX10*	Yes	No	No	1.40E-02	-0.83	Down
ENSG00000063046	*EIF4B*	Yes	No	No	1.41E-02	0.41	Up
ENSG00000255468	*AP001107.9*	Yes	No	No	1.43E-02	-0.41	Down

ENSG00000114353	*GNAI2*	Yes	No	No	1.43E-02	-0.50	Down
ENSG00000163866	*SMIM12*	Yes	No	No	1.45E-02	-0.59	Down
ENSG00000072756	*TRNT1*	Yes	No	No	1.47E-02	0.23	Up
ENSG00000241111	*AC092040.1*	Yes	No	No	1.47E-02	0.96	Up
ENSG00000168487	*BMP1*	Yes	No	No	1.47E-02	-1.04	Down
ENSG00000102710	*SUPT20H*	Yes	No	No	1.48E-02	0.32	Up
ENSG00000074219	*TEAD2*	Yes	No	No	1.50E-02	-0.85	Down
ENSG00000254614	*AP003068.2*	Yes	No	No	1.50E-02	-0.58	Down
ENSG00000248668	*OXCT1-AS1*	Yes	No	No	1.52E-02	0.54	Up
ENSG00000225602	*MTOR-AS1*	Yes	No	No	1.53E-02	-0.27	Down
ENSG00000174059	*CD34*	Yes	No	No	1.53E-02	-0.69	Down
ENSG00000132205	*EMILIN2*	No	No	No	1.53E-02	-0.95	Down
ENSG00000250565	*ATP6V1E2*	Yes	No	No	1.54E-02	0.64	Up
ENSG00000196230	*TUBB*	Yes	No	No	1.54E-02	-0.59	Down
ENSG00000180900	*SCRIB*	Yes	No	No	1.54E-02	-0.57	Down
ENSG00000150995	*ITPR1*	Yes	No	No	1.55E-02	0.54	Up
ENSG00000151640	*DPYSL4*	Yes	No	No	1.58E-02	-0.80	Down
ENSG00000125753	*VASP*	Yes	No	No	1.61E-02	-0.65	Down
ENSG00000269934	*AL353593.2*	Yes	No	No	1.61E-02	0.37	Up
ENSG00000113749	*HRH2*	No	No	Yes	1.62E-02	-0.74	Down
ENSG00000163346	*PBXIP1*	Yes	No	No	1.62E-02	-0.45	Down
ENSG00000280239	*AC011498.7*	Yes	No	No	1.64E-02	0.24	Up
ENSG00000103316	*CRYM*	No	No	No	1.65E-02	0.88	Up
ENSG00000224934	*AL391684.1*	No	No	No	1.65E-02	0.73	Up
ENSG00000154133	*ROBO4*	Yes	No	No	1.65E-02	-0.83	Down
ENSG00000237781	*AL356356.1*	No	No	Yes	1.68E-02	-0.81	Down
ENSG00000198542	*ITGBL1*	No	No	No	1.68E-02	1.02	Up
ENSG00000065717	*TLE2*	Yes	No	No	1.72E-02	-1.17	Down
ENSG00000214193	*SH3D21*	Yes	No	No	1.72E-02	0.43	Up
ENSG00000197766	*CFD*	Yes	No	No	1.72E-02	-0.67	Down
ENSG00000210195	*MT-TT*	Yes	No	No	1.72E-02	-1.18	Down
ENSG00000254027	*AC009902.2*	Yes	No	No	1.72E-02	-0.52	Down
ENSG00000174429	*ABRA*	No	No	No	1.72E-02	-1.02	Down
ENSG00000001167	*NFYA*	Yes	No	No	1.73E-02	1.12	Up
ENSG00000138495	*COX17*	Yes	No	No	1.74E-02	-0.60	Down
ENSG00000172935	*MRGPRF*	No	No	No	1.76E-02	-0.98	Down
ENSG00000161011	*SQSTM1*	Yes	No	No	1.79E-02	0.26	Up
ENSG00000080824	*HSP90AA1*	Yes	No	No	1.81E-02	-0.55	Down
ENSG00000072952	*MRVI1*	No	No	Yes	1.82E-02	-0.77	Down
ENSG00000134460	*IL2RA*	No	No	Yes	1.82E-02	-1.48	Down

ENSG00000138587	*MNS1*	Yes	No	No	1.85E-02	1.38	Up
ENSG00000166816	*LDHD*	Yes	No	No	1.87E-02	-0.63	Down
ENSG00000104825	*NFKBIB*	Yes	No	No	1.88E-02	-0.76	Down
ENSG00000109062	*SLC9A3R1*	Yes	No	No	1.88E-02	-0.87	Down
ENSG00000185818	*NAT8L*	No	Yes	No	1.89E-02	1.16	Up
ENSG00000136717	*BIN1*	Yes	No	No	1.89E-02	-0.83	Down
ENSG00000162191	*UBXN1*	Yes	No	No	1.89E-02	-0.55	Down
ENSG00000138459	*SLC35A5*	Yes	No	No	1.89E-02	0.35	Up
ENSG00000134851	*TMEM165*	Yes	No	No	1.92E-02	-0.53	Down
ENSG00000182162	*P2RY8*	Yes	No	No	1.99E-02	1.07	Up
ENSG00000234438	*KBTBD13*	No	No	Yes	2.00E-02	-0.93	Down
ENSG00000245322	*AC097460.1*	Yes	No	No	2.00E-02	-0.43	Down
ENSG00000258092	*AC005841.1*	Yes	No	No	2.01E-02	-0.47	Down
ENSG00000196154	*S100A4*	No	No	No	2.03E-02	-1.09	Down
ENSG00000172795	*DCP2*	Yes	No	No	2.04E-02	0.83	Up
ENSG00000271806	*AL590822.2*	Yes	No	No	2.06E-02	-0.58	Down
ENSG00000131386	*GALNT15*	No	No	No	2.08E-02	-1.16	Down
ENSG00000255750	*AC022509.1*	No	No	No	2.12E-02	1.14	Up
ENSG00000119917	*IFIT3*	Yes	No	No	2.14E-02	0.92	Up
ENSG00000160075	*SSU72*	Yes	No	No	2.21E-02	-0.64	Down
ENSG00000066422	*ZBTB11*	Yes	No	No	2.24E-02	0.40	Up
ENSG00000084636	*COL16A1*	Yes	No	No	2.25E-02	0.64	Up
ENSG00000141101	*NOB1*	Yes	No	No	2.25E-02	-0.65	Down
ENSG00000159166	*LAD1*	Yes	No	No	2.36E-02	-1.60	Down
ENSG00000285938	*AC072022.2*	Yes	No	No	2.36E-02	-0.94	Down
ENSG00000235448	*LURAP1L-AS1*	Yes	No	No	2.40E-02	0.38	Up
ENSG00000139278	*GLIPR1*	Yes	No	No	2.41E-02	-0.17	Down
ENSG00000285966	*AC007686.4*	No	No	Yes	2.42E-02	-1.74	Down
ENSG00000198517	*MAFK*	No	No	No	2.48E-02	0.68	Up
ENSG00000250303	*AP002884.1*	No	No	Yes	2.49E-02	-0.95	Down
ENSG00000136279	*DBNL*	Yes	No	No	2.50E-02	-0.47	Down
ENSG00000143537	*ADAM15*	No	No	No	2.51E-02	-0.77	Down
ENSG00000134470	*IL15RA*	No	No	No	2.52E-02	-0.92	Down
ENSG00000167984	*NLRC3*	Yes	No	No	2.52E-02	0.41	Up
ENSG00000138079	*SLC3A1*	Yes	No	No	2.56E-02	0.43	Up
ENSG00000130382	*MLLT1*	Yes	No	No	2.57E-02	-0.64	Down
ENSG00000184371	*CSF1*	Yes	No	No	2.57E-02	-0.97	Down
ENSG00000136068	*FLNB*	Yes	No	No	2.61E-02	-0.52	Down
ENSG00000121039	*RDH10*	No	No	Yes	2.62E-02	-0.90	Down
ENSG00000286186	*AC018445.5*	Yes	No	No	2.63E-02	0.45	Up

ENSG00000162889	*MAPKAPK2*	Yes	No	No	2.64E-02	-0.35	Down
ENSG00000187185	*AC092118.1*	Yes	No	No	2.64E-02	0.58	Up
ENSG00000198176	*TFDP1*	Yes	No	No	2.65E-02	-0.91	Down
ENSG00000181104	*F2R*	No	No	Yes	2.68E-02	0.95	Up
ENSG00000136448	*NMT1*	Yes	No	No	2.74E-02	-0.33	Down
ENSG00000002586	*CD99*	Yes	No	No	2.75E-02	-0.61	Down
ENSG00000170458	*CD14*	No	No	No	2.75E-02	-0.88	Down
ENSG00000114120	*SLC25A36*	Yes	No	No	2.79E-02	0.65	Up
ENSG00000184949	*FAM227A*	Yes	No	No	2.79E-02	1.13	Up
ENSG00000124507	*PACSIN1*	No	No	Yes	2.79E-02	-0.88	Down
ENSG00000119718	*EIF2B2*	Yes	No	No	2.80E-02	-0.34	Down
ENSG00000116786	*PLEKHM2*	Yes	No	No	2.81E-02	-0.58	Down
ENSG00000124702	*KLHDC3*	Yes	No	No	2.81E-02	-0.81	Down
ENSG00000188846	*RPL14*	Yes	No	No	2.81E-02	-0.45	Down
ENSG00000176463	*SLCO3A1*	Yes	No	No	2.85E-02	-0.56	Down
ENSG00000065621	*GSTO2*	Yes	No	No	2.88E-02	1.00	Up
ENSG00000260630	*SNAI3-AS1*	Yes	No	No	2.89E-02	-0.97	Down
ENSG00000137161	*CNPY3*	Yes	No	No	2.92E-02	-0.49	Down
ENSG00000182177	*ASB18*	No	No	Yes	2.95E-02	0.92	Up
ENSG00000211455	*STK38L*	Yes	No	No	2.95E-02	0.90	Up
ENSG00000204219	*TCEA3*	Yes	No	No	2.98E-02	-0.54	Down
ENSG00000140395	*WDR61*	Yes	No	No	3.01E-02	0.43	Up
ENSG00000137198	*GMPR*	Yes	No	No	3.06E-02	-0.75	Down
ENSG00000175130	*MARCKSL1*	Yes	No	No	3.11E-02	-0.93	Down
ENSG00000133275	*CSNK1G2*	Yes	No	No	3.22E-02	-0.62	Down
ENSG00000173156	*RHOD*	Yes	No	No	3.24E-02	-1.06	Down
ENSG00000179743	*AL450998.2*	Yes	No	No	3.25E-02	-0.64	Down
ENSG00000232295	*AL589935.1*	Yes	No	No	3.26E-02	0.63	Up
ENSG00000242396	*AC096536.2*	No	No	No	3.26E-02	-1.14	Down
ENSG00000099917	*MED15*	Yes	No	No	3.29E-02	-0.61	Down
ENSG00000101210	*EEF1A2*	Yes	No	No	3.31E-02	-0.41	Down
ENSG00000068831	*RASGRP2*	Yes	No	No	3.35E-02	-0.96	Down
ENSG00000099785	*MARCH2*	Yes	No	No	3.36E-02	-0.69	Down
ENSG00000092010	*PSME1*	Yes	No	No	3.37E-02	-0.57	Down
ENSG00000203883	*SOX18*	No	No	No	3.39E-02	-0.74	Down
ENSG00000137168	*PPIL1*	No	No	Yes	3.44E-02	-0.54	Down
ENSG00000132481	*AC087289.1*	No	No	No	3.44E-02	-0.74	Down
ENSG00000203865	*ATP1A1-AS1*	Yes	No	No	3.44E-02	-0.47	Down
ENSG00000130921	*C12orf65*	Yes	No	No	3.49E-02	-0.37	Down
ENSG00000183023	*SLC8A1*	Yes	No	No	3.54E-02	0.68	Up

ENSG00000104805	*NUCB1*	Yes	No	No	3.54E-02	-0.44	Down
ENSG00000100564	*PIGH*	Yes	No	No	3.54E-02	-0.48	Down
ENSG00000280064	*AC130304.1*	Yes	No	No	3.54E-02	0.76	Up
ENSG00000124251	*TP53TG5*	Yes	No	No	3.54E-02	-0.49	Down
ENSG00000162458	*FBLIM1*	No	No	No	3.54E-02	-0.78	Down
ENSG00000166441	*RPL27A*	Yes	No	No	3.55E-02	-0.23	Down
ENSG00000196470	*SIAH1*	Yes	No	No	3.59E-02	0.26	Up
ENSG00000204628	*RACK1*	Yes	No	No	3.60E-02	-0.31	Down
ENSG00000170515	*PA2G4*	Yes	No	No	3.60E-02	-0.55	Down
ENSG00000106809	*OGN*	No	No	No	3.61E-02	1.18	Up
ENSG00000130726	*TRIM28*	Yes	No	No	3.68E-02	-0.69	Down
ENSG00000268756	*AC104534.1*	Yes	No	No	3.73E-02	0.78	Up
ENSG00000167994	*RAB3IL1*	Yes	No	No	3.76E-02	-0.96	Down
ENSG00000196923	*PDLIM7*	No	No	No	3.76E-02	-0.98	Down
ENSG00000064726	*BTBD1*	Yes	No	No	3.78E-02	0.43	Up
ENSG00000131469	*RPL27*	Yes	No	No	3.79E-02	-0.66	Down
ENSG00000108774	*RAB5C*	Yes	No	No	3.80E-02	-0.78	Down
ENSG00000127774	*EMC6*	Yes	No	No	3.83E-02	-0.60	Down
ENSG00000132359	*RAP1GAP2*	Yes	No	No	3.83E-02	-0.77	Down
ENSG00000267801	*AC087289.6*	Yes	No	No	3.90E-02	-0.70	Down
ENSG00000116106	*EPHA4*	Yes	No	No	3.92E-02	0.66	Up
ENSG00000167797	*CDK2AP2*	No	No	No	3.94E-02	-0.81	Down
ENSG00000125656	*CLPP*	Yes	No	No	3.94E-02	-0.91	Down
ENSG00000221968	*FADS3*	Yes	No	No	3.94E-02	-0.61	Down
ENSG00000027697	*IFNGR1*	Yes	No	No	3.96E-02	-0.38	Down
ENSG00000245330	*AP005717.1*	Yes	No	No	3.98E-02	-0.41	Down
ENSG00000279086	*AC073130.3*	Yes	No	No	3.98E-02	-0.62	Down
ENSG00000160801	*PTH1R*	No	No	No	4.02E-02	-0.87	Down
ENSG00000147155	*EBP*	No	No	No	4.05E-02	-1.18	Down
ENSG00000160789	*LMNA*	Yes	No	No	4.06E-02	-0.47	Down
ENSG00000139567	*ACVRL1*	Yes	No	No	4.08E-02	-0.73	Down
ENSG00000186814	*ZSCAN30*	Yes	No	No	4.12E-02	0.45	Up
ENSG00000101342	*TLDC2*	Yes	No	No	4.13E-02	-0.69	Down
ENSG00000177679	*SRRM3*	No	No	No	4.18E-02	-1.07	Down
ENSG00000106823	*ECM2*	No	No	No	4.22E-02	1.00	Up
ENSG00000149596	*JPH2*	Yes	No	No	4.24E-02	-0.51	Down
ENSG00000177156	*TALDO1*	Yes	No	No	4.27E-02	-0.60	Down
ENSG00000269982	*AC018809.2*	Yes	No	No	4.32E-02	-0.19	Down
ENSG00000070540	*WIPI1*	Yes	No	No	4.32E-02	0.37	Up
ENSG00000173272	*MZT2A*	No	No	No	4.33E-02	-0.66	Down

ENSG00000227051	*C14orf132*	No	No	Yes	4.34E-02	0.91	Up
ENSG00000140988	*RPS2*	Yes	No	No	4.35E-02	-0.71	Down
ENSG00000187051	*RPS19BP1*	Yes	No	No	4.39E-02	-0.56	Down
ENSG00000063177	*RPL18*	Yes	No	No	4.40E-02	-0.51	Down
ENSG00000106819	*ASPN*	No	No	No	4.40E-02	1.27	Up
ENSG00000159884	*CCDC107*	Yes	No	No	4.40E-02	-0.61	Down
ENSG00000135624	*CCT7*	Yes	No	No	4.42E-02	-0.53	Down
ENSG00000101400	*SNTA1*	Yes	No	No	4.44E-02	-0.57	Down
ENSG00000110987	*BCL7A*	Yes	No	No	4.50E-02	0.44	Up
ENSG00000141404	*GNAL*	Yes	No	No	4.51E-02	0.11	Up
ENSG00000182718	*ANXA2*	Yes	No	No	4.55E-02	-0.96	Down
ENSG00000108518	*PFN1*	Yes	No	No	4.58E-02	-0.54	Down
ENSG00000235505	*CASP17P*	Yes	No	No	4.58E-02	1.23	Up
ENSG00000116717	*GADD45A*	Yes	No	No	4.63E-02	0.71	Up
ENSG00000165886	*UBTD1*	Yes	No	No	4.66E-02	-0.87	Down
ENSG00000100523	*DDHD1*	Yes	No	No	4.67E-02	0.70	Up
ENSG00000130309	*COLGALT1*	Yes	No	No	4.71E-02	-0.82	Down
ENSG00000180999	*C1orf105*	No	No	No	4.76E-02	-1.41	Down
ENSG00000198663	*C6orf89*	Yes	No	No	4.77E-02	-0.37	Down
ENSG00000148175	*STOM*	Yes	No	No	4.79E-02	-0.56	Down
ENSG00000170935	*NCBP2L*	Yes	No	No	4.81E-02	0.61	Up
ENSG00000149554	*CHEK1*	Yes	No	No	4.82E-02	0.62	Up
ENSG00000172348	*RCAN2*	Yes	No	No	4.84E-02	-0.47	Down
ENSG00000175287	*PHYHD1*	No	No	No	4.85E-02	-0.87	Down
ENSG00000279296	*PRAL*	No	No	No	4.88E-02	0.50	Up
ENSG00000170801	*HTRA3*	Yes	No	No	4.89E-02	-0.55	Down
ENSG00000138622	*HCN4*	Yes	No	No	4.90E-02	-1.16	Down
ENSG00000187955	*COL14A1*	No	No	No	4.90E-02	0.93	Up
ENSG00000055070	*SZRD1*	Yes	No	No	4.91E-02	-0.43	Down
ENSG00000134684	*YARS*	No	No	No	4.91E-02	-0.68	Down
ENSG00000132938	*MTUS2*	Yes	No	No	4.91E-02	-0.58	Down
ENSG00000248636	*AC002070.1*	Yes	No	No	4.93E-02	0.73	Up
ENSG00000228804	*AC072022.1*	No	No	No	4.96E-02	-0.71	Down
ENSG00000087074	*PPP1R15A*	Yes	No	No	4.97E-02	0.57	Up
ENSG00000137492	*THAP12*	Yes	No	No	4.97E-02	0.98	Up
ENSG00000186185	*KIF18B*	Yes	No	No	4.99E-02	-2.00	Down

1: Indicates if the gene was differentially expressed in each individual study; 2: FDR-adjusted p-value from meta-analysis, “E” represents “times ten raised to the power of”; 3: Average of log_2FC from individual studies; 4: ‘Up’ or ‘Down’ indicates if the gene was upregulated or downregulated. The genes were sorted based on the adjusted p-values.

Supplementary Table S2. Differentially expressed upstream regulators and their target genes.

Upstream Regulator	Log_2FC	Molecule Type	Activation z-score[1]	p-value of overlap[2]	Target Molecules in Dataset
SMARCA4	-0.51	transcription regulator	-3.00	3.51E-07	*ACTA2, ADAM15, BIN1, C1orf105, C1orf54, CKM, CSF1, CSNK1G2, CTSB, DES, ECM2, EMP3, EPHA4, FADS3, FBLIM1, FLNB, GADD45A, GYS1, HLA-B, IER3, IFITM2, IL15RA, ITGA7, ITPR1, LMNA, LUM, MT-ND2, MYL4, NECTIN1, PCDH1, PLEKHO1, PLIN2, RAD1, RHBDL2, SLC11A1, SMARCA4, STARD10, TGFB2, TNNT2, TPM1, TUBA3C/TUBA3D, TUBB, ZNF503*
ERBB2	-0.58	kinase	-0.86	3.58E-07	*ACTA2, ACTB, ALDOC, ANXA2, ATF4, BCL2L1, BMP1, BMP7, CD34, CDC25B, CDC7, CHEK1, COL4A1, COL6A1, COL6A3, CTSB, CUL3, DAG1, DDX10, EBP, EIF4EBP1, EMP3, ERBB2, ETV4, F2R, FLOT2, GPX3, GTF3A, IGF1R, KLHDC3, LAMP1, LUM, MKNK2, MME, MYL9, NID1, PLIN2, POLR1D, PSMC3, RHOD, S100A4, SLC2A1, ST3GAL6, STAT3, TCEAL9, THBS4, TIMP3, TPM1, USF2*
TFRC	-0.99	transporter	0.19	5.77E-06	*ANXA2, ATF4, F13A1, FAM20C, FTH1, GADD45A, GPX3, PPP1R15A, PTN, SLC2A1, TGFB2, UBE2E1*
THBS4	0.79	other	-1.77	1.74E-04	*ATF4, CALR, CRELD2, HYOU1, P4HB, TGFB2*
IGF1R	0.62	transmembrane receptor	-2.00	4.86E-04	*ACTA2, BCL2L1, CD37, COL4A1, COL4A2, COX6A2, DES, FLNC, FTH1, IGF1R, NDUFB4, RPL37A, RPS24, S100A4, SCARA5, STAT3, TFDP1, TIMP3, TPO*
PMP22	-0.86	other		7.98E-04	*EMP3, FLOT1, NGFR, PMP22*
ATF4	-0.77	transcription regulator	-1.09	1.03E-03	*ATF4, CALR, EIF4EBP1, GADD45A, GYS1, HYOU1, MAP1LC3B, MID1IP1, NUPR1, PMP22, PPP1R15A, STAT3, TGFB2*
ATG7	0.26	enzyme	0.32	1.39E-03	*ACTA2, ATF4, CTSB, LAMP1, MARCO, S100A4, SLC2A1, SQSTM1*
ACTG1	-0.73	other		1.89E-03	*ACTA2, ACTB, CNN1*
DAG1	-0.70	transmembrane receptor	-1.00	2.06E-03	*DAG1, ITGA7, SLC2A1, SNTA1*
PDCD5	-0.99	other		2.57E-03	*ATP2A2, FOXP3*
PDGFRB	-0.97	kinase		5.90E-03	*ACTA2, PDGFRB, STAT3*
PFN1	-0.54	other		8.23E-03	*ANXA2, SQSTM1*
S100A9	-2.13	other	0.65	1.20E-02	*ADAMTS4, ALPL, C1QB, C3, COX6A2, FBLIM1, FURIN, NGFR, PHLDA1, S100A9, SOCS7*
CLPP	-0.91	peptidase	2.45	1.24E-02	*IDH2, IDH3G, OGDH, PRDX3, SLC25A4, TALDO1*

NCAM1	-0.61	other		1.29E-02	*CALR, NCAM1, NFKBIB*
NFYA	1.12	transcription regulator		1.46E-02	*CALR, CDC25B, FTH1, GADD45A, PDGFRB, PPP1R15A, THRSP*
TGFB2	1.13	growth factor	-0.73	1.57E-02	*ACTA2, ASPN, CNN1, DES, FMOD, FURIN, PDGFRB, TGFB2*
PTP4A3	-0.94	phosphatase		1.75E-02	*HSP90AA1, IGF1R, PCBP1*
DNMT1	-0.87	enzyme		1.83E-02	*ACTB, CMTM5, DNMT1, FOXP3, PTN, SFRP4, TIMP3*
PPP1R15A	0.57	other		2.02E-02	*ATF4, GADD45A, MAP1LC3B*
ETV4	1.28	transcription regulator		2.11E-02	*ACTA2, CAV1, ERBB2, ETV4*
EIF4G1	-0.80	translation regulator		2.61E-02	*BCL2L1, CHEK1, GADD45A*
TPO	-1.15	enzyme		2.96E-02	*FOXP3*
MPP3	-1.09	kinase		2.96E-02	*NECTIN1*
CFD	-0.68	peptidase		2.96E-02	*C3*
TNNC1	-0.57	other		2.96E-02	*SLC8A1*
JPH2	-0.51	enzyme		2.96E-02	*SLC8A1*
EIF2B2	-0.34	other		2.96E-02	*ACTB*
FMOD	1.13	other		2.96E-02	*LUM*
FTH1	-0.72	enzyme		3.36E-02	*FTH1, TFRC*
LY6K	1.57	other		3.36E-02	*CD34, LY6K*
HIST1H1C	-0.80	other		4.03E-02	*ACTB, IER3*
SGPP2	-1.46	phosphatase		4.04E-02	*ATF4, HSP90AA1, HSPB7*
CTSB	-0.50	peptidase		4.45E-02	*ACTA2, BCL2L1, CTSB*
MBD2	-0.80	transcription regulator	0.15	4.56E-02	*ACTB, ADAM15, FOXP3, NUPR1, RNASE1*
STK40	-0.71	kinase		4.66E-02	*C1QA, C1QB, CKM, DES*
TAF6	-0.23	transcription regulator		4.88E-02	*GADD45A, SRSF4, UNC45A*

1: Positive and negative z-scores are considered as activated and inhibited, respectively; 2: p-value < 0.05 reflects a significant association between the upstream regulator and its targeted genes, “E” represents “times ten raised to the power of”. The genes were sorted based on the p-value of overlap.

Supplementary Table S3. Significant canonical pathways with absolute z-score > 2.0 and the involved genes.

Ingenuity Canonical Pathways	-log(p)[1]	z-score[2]	genes
EIF2 Signaling	12.4	-2.83	*ACTA2, ACTB, ACTC1, ATF4, EIF2B2, EIF4G1, IGF1R, MAP2K2, PPP1R15A, PTBP1, RPL14, RPL18, RPL23, RPL26, RPL27, RPL27A, RPL35, RPL37A, RPL6, RPL8, RPLP0, RPLP1, RPLP2, RPS12, RPS18, RPS2, RPS24, RPS25, RPS4X, RPS5, RPS9*
RhoA Signaling	4.27	-2.89	*ACTA2, ACTB, ACTC1, ACTG1, CDC42EP4, IGF1R, MYL3, MYL4, MYL7, MYL9, PFN1, PKN1, PLXNA1*
Integrin Signaling	3.8	-3.15	*ACTA2, ACTB, ACTC1, ACTG1, CAPN1, CAPN10, CAPNS1, CAV1, ITGA7, MAP2K2, MYL7, MYL9, PARVB, PFN1, RAPGEF1, RHOD, VASP*
Actin Cytoskeleton Signaling	3.68	-3.21	*ACTA2, ACTB, ACTC1, ACTG1, CD14, F2R, FGF14, FLNA, MAP2K2, MYH6, MYH9, MYL3, MYL4, MYL7, MYL9, PFN1, TMSB10/TMSB4X*
Cardiac Hypertrophy Signaling (Enhanced)	3.39	-2.27	*ADCY3, ADRB1, ATP2A2, CACNA1D, EIF2B2, EIF4EBP1, FGF14, FICD, GDE1, GNA11, GNAI2, HSPB7, IGF1R, IL15RA, IL2RA, ITPR1, MAP2K2, MAP3K6, MAPK10, MAPKAPK2, MKNK2, NFATC4, NGFR, NKX2-5, PKN1, PLCE1, STAT3, TGFB2*
Complement System	3.23	-2.45	*C1QA, C1QB, C1QC, C1R, C3, CFD*
Signaling by Rho Family GTPases	3.16	-3.21	*ACTA2, ACTB, ACTC1, ACTG1, CDC42EP4, DES, GNA11, GNAI2, GNAL, MAP2K2, MAPK10, MYL3, MYL4, MYL7, MYL9, PKN1, RHOD*
Regulation of Actin-based Motility by Rho	2.82	-3.00	*ACTA2, ACTB, ACTC1, MYL3, MYL4, MYL7, MYL9, PFN1, RHOD*
RhoGDI Signaling	2.23	3.00	*ACTA2, ACTB, ACTC1, ACTG1, GNA11, GNAI2, GNAL, MYL3, MYL4, MYL7, MYL9, RHOD*
VEGF Signaling	1.6	-2.45	*ACTA2, ACTB, ACTC1, ACTG1, BCL2L1, EIF2B2, MAP2K2*
Ephrin Receptor Signaling	1.45	-2.12	*ATF4, EPHA4, EPHB1, GNA11, GNAI2, GNAL, ITSN1, MAP2K2, RAPGEF1, STAT3*

1: -log(p) > 1.3 reflects a significant association between the canonical pathway and its involved genes; 2: Positive and negative z-scores are considered as activated and inhibited, respectively. The pathways were sorted based on the -log(p).
